# Magnetic Resonance Imaging–Based Artificial Intelligence in Predicting Prostate Cancer Biochemical Recurrence: Systematic Review and Meta-Analysis

**DOI:** 10.2196/85360

**Published:** 2026-07-07

**Authors:** Yanjun Jin, Tianzuo Yuan, Zhiyuan Chen

**Affiliations:** 1 Faculty of Health Sciences, University of Macau, Avenida da Universidade, Macau, 999078, China; 2 Department of Urology, Renmin Hospital of Wuhan University, 99 Zhang Zhi-dong Road, Wuhan, Hubei, 430060, P.R. China, 86 13886000807; 3 Institute of Urologic Disease, Renmin Hospital of Wuhan University, Wuhan, Hubei, China

**Keywords:** artificial intelligence, prostate cancer, predict, biochemical recurrence, meta-analysis

## Abstract

**Background:**

Artificial intelligence (AI) has emerged as a promising tool for prostate cancer (PCa) risk stratification and outcome prediction. However, current studies often lack multicenter external validation, have limited sample sizes, present significant intermodel variability, and face overfitting concerns.

**Objective:**

This study aimed to comprehensively evaluate the diagnostic performance of magnetic resonance imaging (MRI)–based AI models in predicting biochemical recurrence (BCR) of PCa.

**Methods:**

Systematic searches were conducted in the PubMed, Embase, Web of Science, and Cochrane Library databases up to January 13, 2026. Studies were included that involved participants diagnosed with PCa, used MRI-based AI for predicting BCR, and had clearly defined reference standards. The quality of the included studies was assessed using the PROBAST+AI tool. A bivariate random effects model was used to pool sensitivity, specificity, and area under the curve (AUC) statistics.

**Results:**

A total of 28 studies were included, with 2623 patients in internal validation and 1134 patients in external validation. Diagnostic contingency tables were reconstructed from published performance metrics for most studies, while others were extracted from receiver operating characteristic curves due to the lack of direct reporting. In the internal validation set, pooled sensitivity was 0.80 (95% CI 0.73‐0.86; prediction interval [PI] 0.48‐0.99), specificity was 0.83 (95% CI 0.77‐0.89; PI 0.49‐1.00), and AUC was 0.86 (95% CI 0.83‐0.89; PI 0.74‐0.99). In the external validation set, pooled sensitivity was 0.82 (95% CI 0.72‐0.91; PI 0.54‐0.99), specificity was 0.83 (95% CI 0.71‐0.92; PI 0.49‐1.00), and AUC was 0.84 (95% CI 0.79‐0.90; PI 0.70‐0.98). No statistically significant differences were observed between internal and external validation in sensitivity (*P*=.73), specificity (*P*>.99), AUC (*P*=.53), or diagnostic odds ratio (*P*=.98). Medical Net and Extreme Gradient Boosting achieved the highest sensitivity and AUC, whereas multiple kernel learning and support vector machine had the highest specificity. Subgroup and meta-regression analyses suggested that AI method, model type, timing of MRI acquisition, and treatment modality may contribute to heterogeneity.

**Conclusions:**

This meta-analysis innovatively realizes the quantitative direct comparison of MRI-based AI model performance between internal and external validation cohorts for PCa BCR prediction. It comprehensively evaluates AI performance across diverse PCa treatment modalities and integrates machine learning and deep learning approaches. For the field, it identifies key sources of performance heterogeneity (eg, MRI acquisition timing and treatment modality) and quantifies the sensitivity-specificity trade-off in integrated radiomic-clinical models, advancing the systematic understanding of MRI-based AI for BCR prediction. In real-world practice, it provides actionable guidance to prioritize pretreatment MRI for AI model development and clinical BCR assessment and underscores the urgent need for standardized imaging protocols and prospective multicenter studies, laying a foundation for the safe clinical translation of these AI tools as adjunctive decision support instruments.

## Introduction

Prostate cancer (PCa) is a prevalent malignancy among men worldwide, with a rising incidence [1]. In 2025, approximately 313,780 new cases of prostate cancer and 35,770 deaths are expected in the United States alone, highlighting its ongoing impact on public health [2]. Globally, PCa continues to represent 14.1% of all cancer cases and 6.8% of cancer-related mortality, with the number of new diagnoses and deaths steadily increasing in low- and middle-income regions [3]. While radical prostatectomy (RP) and radiation therapy (RT) serve as primary treatments, a subset of intermediate- and high-risk patients still experience biochemical recurrence (BCR) following therapy, typically defined by a sustained increase in prostate-specific antigen (PSA) levels [4,5]. Patients who experience BCR carry significantly worse prognoses, with elevated risks of metastasis and mortality [1]. Therefore, early and precise identification of individuals at high risk of BCR is paramount for guiding clinical management, tailoring interventions, and improving quality-adjusted survival outcomes.

Current clinical detection of PCa relies on PSA testing, bone scans, multiparametric magnetic resonance imaging, prostate-specific membrane antigen positron emission tomography and computed tomography imaging techniques, and transrectal ultrasound–guided biopsy [5]. While PSA testing is convenient, its specificity is limited [6]; transrectal ultrasound–guided biopsy is prone to sampling errors and observer subjectivity [7,8]; and multiparametric magnetic resonance imaging and prostate-specific membrane antigen Positron Emission Tomography and Computed Tomographypositron emission tomography offer superior localization and risk stratification but remain susceptible to interreader variability and qualitative interpretation bias [9]. Critically, these modalities fail to reliably extract high-dimensional quantitative imaging features, limiting their ability to reveal latent biological information [5,10].

In recent years, artificial intelligence (AI) has emerged as a promising tool in PCa diagnosis. AI outperforms traditional methodologies in processing complex datasets, uncovering hidden nonlinear relationships, and improving predictive accuracy and stability [1,4]. Magnetic resonance imaging (MRI)–based AI models integrating radiomics and deep learning have shown promising potential in predicting BCR after prostatectomy or other treatments. For instance, Hou et al [11] reported a biopsy-free AI-aided precision MRI model in the *British Journal of Cancer*, demonstrating robust area under the curve (AUC) values in both internal and external validation cohorts. However, current studies are mostly retrospective and exploratory in nature, with limited sample sizes, multicenter external validation deficiency, significant intermodel variability, and overfitting concerns [12]. These MRI-based AI models are intended for key clinical use cases, including pretreatment BCR risk stratification, posttreatment surveillance triage, and decision support for salvage therapy, yet their generalizability, robustness, and clinical applicability remain to be firmly established [13,14].

Several systematic reviews and meta-analyses have explored the value of AI in predicting PCa BCR. A recent systematic review by Liu et al [1] overviewed AI applications for predicting BCR following RP, highlighting that MRI-based AI models achieved the highest median AUC–receiver operating characteristic (ROC) compared with models using pathological or clinical-pathological variables alone; however, this review did not perform meta-analytic synthesis or evaluate the influence of different treatment modalities. Furthermore, a meta-analysis by Salimi et al [5] pooled data from MRI-based radiomics models for BCR prediction and reported pooled diagnostic performance, but this study only focused on radiomics-only models and did not stratify or compare model performance between internal and external validation cohorts.

To address these critical gaps, this systematic review and meta-analysis aimed to comprehensively evaluate the diagnostic performance of MRI-based AI models for predicting BCR in PCa, with a direct comparison of performance differences between internal and external validation cohorts and quantification of overall performance and clinical feasibility. These innovations fill key research vacancies and provide robust evidence for the clinical translation of MRI-based AI tools in PCa BCR prediction.

## Methods

### Overview

This meta-analysis adhered rigorously to the PRISMA-DTA (Preferred Reporting Items for Systematic Reviews and Meta-Analyses of Diagnostic Test Accuracy) [15] reporting guidelines and the PRISMA-S extension for reporting literature searches [16]. The completed PRISMA 2020 Abstract, PRISMA-DTA, and PRISMA-S checklists are provided inChecklist1^3^. In addition, data extraction and critical appraisal of included studies were guided by the CHARMS (Checklist for Critical Appraisal and Data Extraction for Systematic Reviews of Prediction Modelling Studies) framework [17]. A detailed domain-by-domain mapping of the CHARMS to the corresponding sections of this manuscript is provided in Checklist 4. This review has been registered on the website of the International Prospective Register of Systematic Reviews (PROSPERO) under the registration number CRD420251102879.

### Search Strategy

The search was conducted across 4 electronic databases (PubMed, Embase, Web of Science, and Cochrane Library) and supplemented by website searches, citation tracking, and manual searches of the reference lists of included studies. No language or publication date restrictions were applied. No study registries were searched. No additional studies were sought by contacting authors or experts. Two independent reviewers (YJ and TY) conducted the initial screening of titles and abstracts, followed by full-text assessment. The initial search was restricted to publications up to March 18, 2025. An updated search was subsequently performed on January 13, 2026, and a second supplementary search was finalized on March 27, 2026. The search strategy consisted of 3 keyword groups: the first group included terms related to AI (eg, “Artificial Intelligence,”, “Machine Learning,” and “Deep Learning”); the second group comprised terms pertaining to the target outcome (eg, “biochemical recurrence” and “Recurrence”); and the third group involved disease-related terms (“Prostate Cancer,”, “Prostate Carcinoma,” and “Prostatic Neoplasms”). A combined approach of free-text terms and Medical Subject Headings (MeSH) was adopted. The search strategies were developed independently and not adapted from prior reviews. No formal peer review of the search strategy was conducted. Detailed specifications of the search strategies are provided in Table S1 in Multimedia Appendix 1.

### Inclusion and Exclusion Criteria

Inclusion criteria followed the PITROS framework. Participants (P) comprised patients with pathologically confirmed PCa, including those undergoing RP, RT, and hormone therapy (HT). Index test (I) involved MRI-based AI models predicting BCR in PCa patients. Reference standard (R) included American Urological Association (AUA) definition, Phoenix definition, Castration-Resistant Prostate Cancer definition, or study-specific BCR definitions [18]. Target condition (T) divided into BCR-positive and BCR-negative groups. Outcomes (O) included sensitivity, specificity, diagnostic odds ratio (DOR), and AUC for internal and external validation sets. Setting (S) encompassed retrospective or prospective studies using public database or institutional data. Notably, the inclusion of multiple definitions aligns with prior systematic reviews in this field [19]. Subgroup analyses by treatment modality were subsequently performed to indirectly address the potential heterogeneity introduced by different BCR criteria, as treatment type is closely correlated with the selection of BCR definition in clinical studies.

Exclusion criteria included (1) irrelevant titles and abstracts; (2) ineligible types (reviews, case reports, conference abstracts, meta-analyses, and letters); (3) studies not focusing on BCR, non-MRI-based AI, or lacking outcome data (true positives [TP], false positives [FP], false negatives [FN], and true negatives [TN]); and (4) overlapping patient cohorts. Duplicates were removed using EndNote X20. Disagreements were resolved by discussion.

### Quality Assessment

The updated PROBAST+AI tool [20], which superseded PROBAST 2019, was used for quality assessment. This tool comprises 2 phases: model development and model evaluation, each encompassing 7 domains that cover participants, data sources, predictors, outcome assessment, and analyses. Each domain is categorized into low (L), high (H), or unclear (U) risk of bias based on responses to specific signaling questions. These questions are rated as “yes” (Y), “probably yes” (PY), “probably no” (PN), “no” (N), “no information” (NI), or “not applicable” (NA) where appropriate. A “yes” or “probably yes” rating indicates low bias risk, while “no” or “probably no” suggests potential high bias risk. Domains with “no information” but “no” or “probably no” ratings are classified as unclear. Detailed signaling questions and assessment tables are available in Tables S2 and S3 in Multimedia Appendix 1. To ensure objectivity and accuracy, 2 reviewers (YJ and TY) independently conducted comprehensive bias risk assessments. Discrepancies during review were resolved by consulting the third reviewer (ZC).

### Data Extraction

Data extraction from eligible full-text articles was conducted independently by 2 reviewers (YJ and TY) to confirm their potential qualification. Discrepancies were resolved through arbitration by a third reviewer (ZC) to reach consensus. Extracted data included patient and study-level information: author; year; country; study design; MRI sequence; treatment; reference standard; analysis approach; and details of training, internal validation, and external validation sets (number of patients per set and count of BCR cases). Technical information encompassed AI method; MRI acquisition time; optimal AI algorithms; optimal AI models; and counts of TP, FP, FN, and TN in validation sets. For studies included in the systematic review but lacking data for meta-analysis, we contacted corresponding authors via email to request missing data.

As most studies did not provide diagnostic contingency tables, 2 strategies were used to reconstruct them: first, back-calculating TP, FP, FN, and TN using sensitivity, specificity, the number of positives per reference standard, and total patient count; second, replotting data via GetData software from ROC curve analyses, extracting optimal sensitivity and specificity based on the maximum Youden index, then back-calculating the 4 metrics using the reference standard-derived positives and total patient numbers. This data reconstruction approach is consistent with established methodological standards in diagnostic meta-analyses, where 2×2 table reconstruction from published performance metrics is commonly used when raw data are unavailable [21].

### Outcome Measures

The primary outcome measures included overall sensitivity, specificity, DOR, and AUC in internal and external validation sets. The secondary outcomes comprised sensitivity, specificity, and AUC stratified by specific algorithms and treatment modalities. Sensitivity (also termed recall or TP rate), reflecting the ability of MRI-based AI models to correctly identify true BCR-positive cases, was calculated as follows: TP/(TP+FN). Specificity (TN rate), indicating the model’s capacity to correctly identify BCR-negative cases, was computed as follows: TN/(TN+FP). AUC served as a composite metric for evaluating the model’s discriminative ability between positive and negative cases. DOR, a comprehensive diagnostic performance index integrating sensitivity and specificity, was defined as the ratio of the odds of a positive test result in BCR-positive patients to that in BCR-negative patients. For studies providing multiple nonoverlapping datasets (eg, 2 external validation sets [22]), contingency tables were treated as independent and fully extracted. For studies reporting multiple AI algorithms, only the algorithm with the highest AUC was included.

### Statistical Analysis

Meta-analyses were performed using a random effects model based on the restricted maximum likelihood method with the Hartung-Knapp-Sidik-Jonkman adjustment [23]. The pooled AUC was calculated. Z-tests were applied to compare diagnostic performance between internal and external validation sets, with statistical significance defined as *P*<.05. Prespecified subgroup analyses were conducted by algorithm type and treatment modality, visualized using violin plots.

Prediction intervals (PIs) and τ² were used to quantify heterogeneity and interpret the weighted pooled average estimate of our meta-analysis in a real-world context [24]. Heterogeneity was also assessed using Higgins’ *I*² statistic, which complements τ² in describing the extent of between-study heterogeneity [25]. For internal validation sets with a sample size >10, bivariate boxplots were used to identify outliers, and subgroup analyses and meta-regression were performed to explore potential sources of heterogeneity. Variables included in subgroup analyses and meta-regression were analysis, AI method, AI model, MRI sequence, time to acquire MRI image, and treatment. Additionally, bubble plots evaluated changes in AI model DOR over time, and radar charts illustrated algorithm distribution in machine learning and deep learning. Fagan plots assessed clinical applicability, using the median BCR positivity rate among included studies as the prior probability. Deeks’ funnel plot asymmetry test was applied to evaluate small-study effects via regression of log DOR against effective sample size; a *P*<.05 for the slope coefficient indicated asymmetry and potential small-study effects [26]. Statistical analyses were performed using the Midas and Metadta packages in Stata 15.1 and the ggplot2 package in R 4.3.2.

### Ethical Considerations

This study was a systematic review and meta-analysis of previously published research; therefore, ethics approval and participant consent were not required.

## Results

### Study Selection

**Figure 1.**
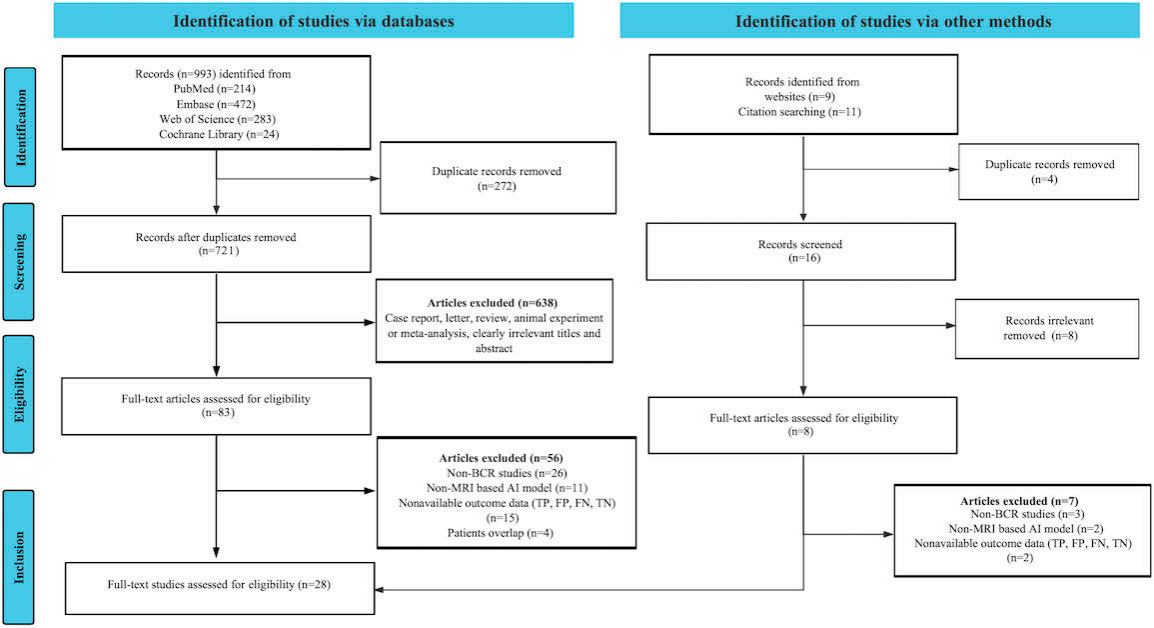
PRISMA (Preferred Reporting Items for Systematic Reviews and Meta-Analyses) flow diagram of study selection. This presents the PRISMA-compliant flow diagram detailing the stepwise study selection process for this systematic review and meta-analysis. AI: artificial intelligence; BCR: biochemical recurrence; FN: false negative; FP: false positive; MRI: magnetic resonance imaging; TN: true negative; TP: true positive.

A comprehensive literature search was conducted to identify potential studies relevant to the research topic. Initially, 993 potentially relevant records were retrieved from 4 primary databases, and 20 additional records were obtained from other nondatabase sources. After removing 272 duplicate records from the database search and 4 duplicate records from other methods, the remaining 721 database records and 16 nondatabase records underwent preliminary screening. During the preliminary screening, 638 database records were excluded due to obvious irrelevance (based on titles or abstracts) or inappropriate study types (case reports, letters, reviews, animal experiments, or meta-analyses), and 8 nondatabase records were removed as irrelevant, leaving 91 articles for full-text review. Upon detailed examination of the full texts, 29 studies were excluded for being non-BCR studies, 13 studies because they did not use MRI-based AI models, 17 studies because of insufficient or incomplete diagnostic data (including TP, FP, FN, and TN) that failed to meet the criteria, and another 4 studies due to patient overlap. Eventually, a total of 28 studies met the inclusion criteria and were included in the meta-analysis [11,18,22,27-51]. The literature screening process adhered to the PRISMA guidelines and is presented in detail in Figure 1.

### Study Description and Quality Assessment

Of the 28 studies included, 25 incorporated internal validation sets [11,18,27-39,41-47,49-51], encompassing a total of 2623 patients, while 7 included external validation with 8 datasets [22,39,40,46-48,50], involving 1134 patients in total. Geographically, the majority (13/28) of studies were conducted in China [11,22,28,32,41,43-48,50,51]. In terms of treatment modalities, 17 studies focused on patients who underwent RP [11,18,22,28,30,32-35,39,40,43,46-49,51], and 11 studies involved patients receiving RT with or without HT [27,29,31,36-39,41,42,44,45]. Among studies involving RT with or without HT, 3 studies specifically used external beam radiation therapy (EBRT) alone [27,29,31], 3 studies used EBRT combined with HT [37,38,44], and 1 study used brachytherapy [45]. In terms of study design, 26 studies were retrospective [11,18,22,28,29,31-51], with only 2 being prospective [27,30]. For the definition of BCR, 16 studies adopted the AUA definition as the gold standard [11,22,28,30,32-35,40,43,46-51], 9 studies used the Phoenix definition [27,29,31,36-38,42,44,45], 1 study simultaneously applied both the Phoenix definition and the Castration-Resistant Prostate Cancer definition [41], 1 study applied both the AUA definition and the Phoenix definition [39], and 1 study used a self-defined standard of PSA ≥0.1 ng/mL [18]. Regarding the analysis method, most studies (26/28) were patient based [11,18,22,27-30,32-51], with only 2 being lesion based [31]. In terms of AI methodologies, 19 studies used machine learning [11,18,27-30,32-34,36-40,43-45,50,51], and 9 studies used deep learning [22,31,35,41,42,46-49]. For the timing of MRI acquisition, 24 studies used pretreatment images [11,18,22,28-30,32-36,38-41,43-51], 3 studies used posttreatment images [31,37,42], and 1 study included both pre- and post-treatment images [27]. Regarding the optimal AI model, 10 studies solely used radiomic features [11,22,27,29-31,33,42,44,45], while 18 studies combined radiomic and clinical features [18,28,32,34-41,43,46-51]. A summary of the study, patient, and technical characteristics is provided in Tables1  2.

**Table 1.** Study and patient characteristics of included studies.

Author	Year	Country	Study design	MRI^a^ sequence	Treatment	Reference standard	Analysis	Patients in total			Number of BCR^b^ patients		
								Training	Internal validation	External validation	Training	Internal validation	External validation
Zhang et al [43]	2016	China	Retro	T2WI^c^, DWI^d^, DCE^e^	RP^f^	AUA^g^ definition	PB^h^	205	205	NA^i^	61	61	NA^i^
Shiradkar et al [39]	2018	USA, Finland	Retro	T2WI, DWI, ADC^j^	RP+RT^k^, RP	AUA definition and Phoenix definition	PB	70	70	50	35	35	7
Park et al [35]	2020	Korea	Retro	T2WI, DWI, ADC, DCE	RP	AUA definition	PB	104	104	NA	24	24	NA
Jambor et al [34]	2019	USA	Retro	T2WI, DWI, DCE	RP	AUA definition	PB	91	91	NA	48	48	NA
Yan et al [22]	2021	China	Retro	T2WI	RP	AUA definition	PB	368	NA	117	99	NA	47
Shiradkar et al [40]	2022	USA	Retro	T2WI, ADC	RP	AUA definition	PB	71	NA	62	27	NA	13
Duenweg et al [30]	2023	USA	Pro	T2WI	RP	AUA definition	PB	186	93	NA	28	18	NA
Hou et al [11]	2023	China	Retro	T2WI, DWI, ADC	RP	AUA definition	PB	463	116	NA	137	34	NA
Hu et al [32]	2024	China	Retro	T2WI, DWI, ADC	RP	AUA definition	PB	254	109	NA	59	25	NA
Sanchez Iglesias et al [37]	2023	Spain	Retro	T2WI, DWI, DCE	EBRT+HT	Phoenix definition	PB	128	128	NA	20	20	NA
Wang et al [41]	2024	China	Retro	ADC	EBRT, HT, EBRT+HT	Phoenix definition & CRPC definition	PB	93	38	NA	23	8	NA
An et al [28]	2023	China	Retro	T2WI, DWI, ADC, PWI^l^	RP	AUA definition	PB	144	62	NA	54	23	NA
Huynh et al [33]	2023	USA	Retro	T2WI, ADC	RP	AUA definition	PB	225	26	NA	44	5	NA
Marín Castrillón et al [29]	2019	NA	Retro	T2WI, ADC	EBRT^m^	Phoenix definition	PB	53	53	NA	8	8	NA
Zhong et al [44]	2020	China	Retro	T1WI, T2WI, DWI	EBRT+HT^n^	Phoenix definition	PB	73	18	NA	23	6	NA
Gumus et al [31]	2024	USA	Retro	T2WI, DWI	EBRT	Phoenix definition	LB	36	36	NA	10	10	NA
Poulakis et al [18]	2004	Germany	Retro	T2WI	RP	PSA≥0.1 ng/ml	PB	200	200	NA	73	73	NA
Yilmaz et al [42]	2023	USA	Retro	T2WI, DWI	RT	Phoenix definition	PB	NA	62	NA	NA	46	NA
Piran Nanekaran et al [36]	2024	Canada	Retro	T2WI	EBRT and EBRT+HT	Phoenix definition	PB	120	30	NA	36	10	NA
Septiers et al [38]	2023	France	Retro	T2WI, DWI	EBRT+HT	Phoenix definition	PB	190	64	NA	29	10	NA
Algohary et al [27]	2022	USA	Pro	T2WI, DWI, ADC, DCE	EBRT	Phoenix definition	PB	25	25	NA	4	5	NA
Zhu et al [45]	2023	China	Retro	T2WI	BT	Phoenix definition	PB	67	16	NA	17	2	NA
Zhu et al [51]	2025	China	Retro	T2WI, DWI, ADC	RP	AUA definition	PB	172	172	NA	115	115	NA
Wu et al [50]	2025	China	Retro	T2WI, FS-T2WI, DWI, ADC	RP+HT	AUA definition	PB	545	545	121	97	97	16
Simon et al [49]	2025	USA	Retro	T2WI, ADC, DWI	RP	AUA definition	PB	240	71	NA	69	16	NA
Niu et al [48]	2025	China	Retro	DWI	RP	AUA definition	PB	182	NA	167	24	NA	19
Lian et al [47]	2025	China	Retro	FS-T2WI, DWI	RP	AUA definition	PB	182	182	50	38	38	8
Li et al [46]	2025	China	Retro	T2WI, ADC, DWI, CE-T1WI	RP	AUA definition	PB	249	107	567	71	28	162

a MRI: magnetic resonance imaging.

b BCR: biochemical recurrence.

c T2WI: T2-weighted images.

d DWI: diffusion-weighted images.

e DCE: dynamic contrast-enhanced images.

f RP: radical prostatectomy.

g AUA: American Urological Association.

h PB: patient based.

i NA: not available.

j ADC: apparent diffusion coefficient.

k RT: radiation therapy.

l PWI: perfusion weighted images.

m EBRT: external beam radiation therapy.

n HT: hormone therapy.

**Table 2.** Technical aspects of included studies.

Author	Year	AI^a^ method	Time to acquire MRI^b^ image	Optimal AI algorithms	Optimal AI model	Internal validation sets					External validation sets				
						TP^c^	FP^d^	FN^e^	TN^f^	AUC^g^ (95% CI)	TP	FP	FN	TN	AUC (95% CI)
Zhang et al [43]	2016	ML^h^	Pre^i^	SVM^j^	Radiomic and clinical	56	8	5	136	0.970 (0.936‐0.988)	NA^k^	NA	NA	NA	NA
Shiradkar et al [39]	2018	ML	Pre	JMI^l^ and SVM	Radiomic and clinical	30	4	5	31	0.820 (0.700‐0.930)	5	14	2	29	0.740 (0.615‐0.865)
Park et al [35]	2020	DL^m^	Pre	AEs^n^	Radiomic and clinical	15	27	9	53	0.825 (0.728‐0.922)	NA	NA	NA	NA	NA
Jambor et al [34]	2019	ML	Pre	RLS^o^	Radiomic and clinical	38	15	10	28	0.770 (0.630‐0.910)	NA	NA	NA	NA	NA
Yan et al [22]^p^	2021	DL	Pre	SNN^q^	Radiomic	NA	NA	NA	NA	NA	12	0	4	18	0.811 (0.722‐0.900)
Yan et al [22]^r^	2021	DL	Pre	SNN	Radiomic	NA	NA	NA	NA	NA	25	9	6	43	0.794 (0.718‐0.870)
Shiradkar et al [40]	2022	ML	Pre	RF^s^ and LR^t^	Radiomic and clinical	NA	NA	NA	NA	NA	8	21	5	28	0.750 (0.630‐0.870)
Duenweg et al [30]	2023	ML	Pre	DT^u^	Radiomic	16	5	2	70	0.740 (0.615‐0.865)	NA	NA	NA	NA	NA
Hou et al [11]	2023	ML	Pre	GB^v^	Radiomic	25	17	9	65	0.860 (0.810‐0.900)	NA	NA	NA	NA	NA
Hu et al [32]	2024	ML	Pre	CPHR^w^	Radiomic and clinical	19	11	6	73	0.911 (0.854‐0.969)	NA	NA	NA	NA	NA
Sanchez Iglesias et al [37]	2023	ML	Post^y^	LR	Radiomic and clinical	19	76	1	32	0.800 (0.690‐0.910)	NA	NA	NA	NA	NA
Wang et al [41]	2024	DL	Pre	MN^x^ and XGB^z^	Radiomic and clinical	8	3	0	27	0.954 (0.892‐1.000)	NA	NA	NA	NA	NA
An et al [28]	2023	ML	Pre	LR	Radiomic and clinical	20	5	3	34	0.930 (0.830‐0.980)	NA	NA	NA	NA	NA
Huynh et al [33]	2023	ML	Pre	RF	Radiomic	3	3	2	18	0.780 (0.670‐0.890)	NA	NA	NA	NA	NA
Marín Castrillón et al [29]	2019	ML	Pre	MKL^aa^ and SVM	Radiomic	6	1	2	44	0.972 (0.780‐0.961)	NA	NA	NA	NA	NA
Zhong et al [44]	2020	ML	Pre	Adaboost	Radiomic	3	2	3	10	0.730 (0.450‐1.000)	NA	NA	NA	NA	NA
Gumus et al [31]	2024	DL	Post	MLP^ab^	Radiomic	8	3	2	23	0.870 (0.720‐1.000)	NA	NA	NA	NA	NA
Poulakis et al [18]	2004	ML	Pre	ANN^ac^	Radiomic and clinical	66	15	7	112	0.897 (0.841‐0.977)	NA	NA	NA	NA	NA
Yilmaz et al [42]	2023	DL	Post	DNN^ad^	Radiomic	35	7	11	9	NA	NA	NA	NA	NA	NA
Piran Nanekaran et al [36]	2024	ML	Pre	GB	Radiomic and clinical	8	2	2	18	0.840 (0.805‐0.881)	NA	NA	NA	NA	NA
Septiers et al [38]	2023	ML	Pre	RF	Radiomic and clinical	5	14	5	40	0.740 (0.650‐0.890)	NA	NA	NA	NA	NA
Algohary et al [27]	2022	ML	Pre and post	LR	Radiomic	4	2	1	18	0.890 (0.693‐1.000)	NA	NA	NA	NA	NA
Zhu et al [45]	2023	ML	Pre	LR	Radiomic	2	6	0	8	0.900 (0.665‐1.000)	NA	NA	NA	NA	NA
Zhu et al [51]	2025	ML	Pre	SVM	Radiomic and clinical	45	5	70	52	0.820 (0.762‐0.878)	NA	NA	NA	NA	NA
Wu et al [50]	2025	ML	Pre	LR	Radiomic and clinical	81	85	16	363	0.892 (0.859‐0.924)	13	18	3	87	0.884 (0.815‐0.953)
Simon et al [49]	2025	DL	Pre	RF	Radiomic and clinical	10	9	6	46	0.736 (0.664‐0.808)	NA	NA	NA	NA	NA
Niu et al [48]	2025	ML	Pre	CPHR	Radiomic and clinical	NA	NA	NA	NA	NA	16	16	3	132	0.869 (0.779‐0.923)
Lian et al [47]	2025	DL	Pre	CNN^ae^ and Transformer	Radiomic and clinical	32	17	6	127	0.857 (0.836‐0.894)	6	6	2	36	0.835 (0.818‐0.869)
Li et al [46]	2026	DL	Pre	CNN and Transformer	Radiomic and clinical	23	4	5	75	0.938 (0.884‐0.992)	150	57	12	348	0.935 (0.900‐0.969)

a AI: artificial intelligence.

b MRI: magnetic resonance imaging.

c TP: true positive.

d FP: false positive.

e FN: false negative.

f TN: true negative.

g AUC: area under the curve.

h ML: machine learning.

i Pre: pretreatment.

j SVM: support vector machine.

k NA: not available.

l JMI: joint mutual information maximization.

m DL: deep learning.

n AE: autoencoder.

o RLS: regularized least squares.

p External validation set from Beijing Friendship Hospital.

q SNN: spiking neural network.

r External validation set from Peking University People’s Hospital.

s RF: random forest.

t LR: logistic regression.

u DT: decision tree.

v GB: gradient boosting.

w CPHR: Cox proportional hazards regression.

x MN: Medical Net.

y Post: posttreatment.

z XGB: Extreme Gradient Boosting.

aa MKL: multiple kernel learning.

ab MLP: multilayer perceptron.

ac ANN: artificial neural network.

ad DNN: deep neural network.

ae CNN: convolutional neural network.

**Figure 2.**
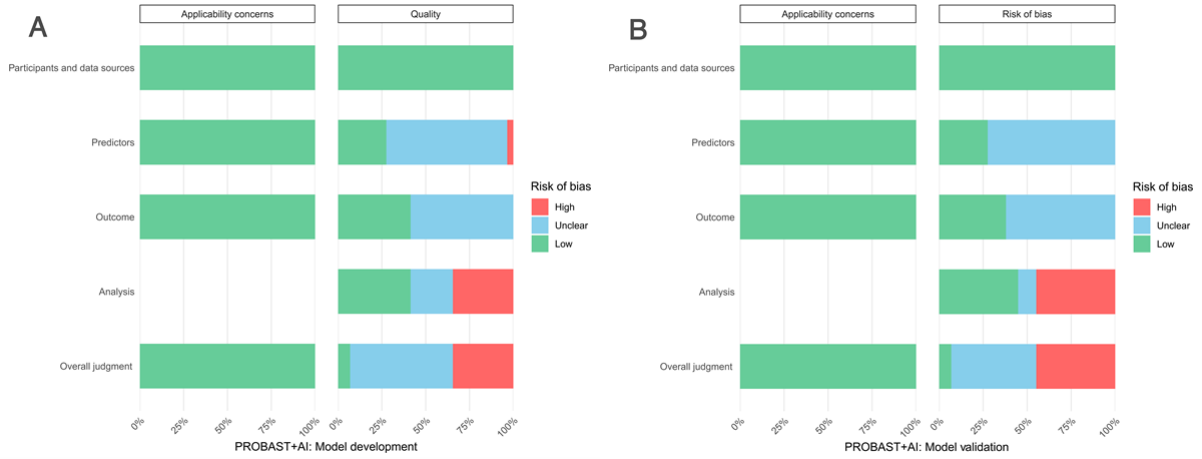
Risk of bias and applicability concerns assessment using PROBAST+AI for included magnetic resonance imaging (MRI)–based AI models. This figure shows the results of risk of bias and applicability concerns assessment using the PROBAST+AI framework for included studies developing and validating MRI-based AI models to predict biochemical recurrence in individuals with pathologically confirmed prostate cancer. (A) Assessment for model development studies, evaluating applicability concerns and overall quality across 4 domains (participants and data sources, predictors, outcome, and analysis) and overall judgment. (B) Assessment for model validation studies, evaluating applicability concerns and risk of bias across the same domains and overall judgment. Each bar represents the proportion of studies rated as low (green), unclear (blue), or high (red) risk of bias or applicability concerns within each domain.

The risk of bias and applicability concerns of all included studies were assessed using the PROBAST+ AI tool. The results of the bias risk assessment for model development and validation are presented in Figure 2, with additional details in Tables S2 and S3 in Multimedia Appendix 1. For model development, in the overall judgment of quality, 36% (10/28) of the studies were rated as having a high risk of bias, 57% (16/28) as having an unclear risk, and the remaining 7% (2/28) as having a low risk. In terms of applicability concerns for model development, all 28 studies (100%) were rated as having low concerns. For model validation, in the overall judgment of risk of bias, 46% (13/28) of the studies were classified as high risk, 46% (13/28) were classified as unclear risk, and 8% (2/28) were classified as low risk. Similarly, all studies (28/28, 100%) were assessed as having low applicability concerns for model validation. While all included studies were rated as having low applicability concerns in both model development and validation, which indicates consistency in study populations and predictors with our research questions, the high and unclear risk of bias in the analysis domains may limit their direct clinical applicability.

### Diagnostic Performance of Different AI Algorithms

**Figure 3.**
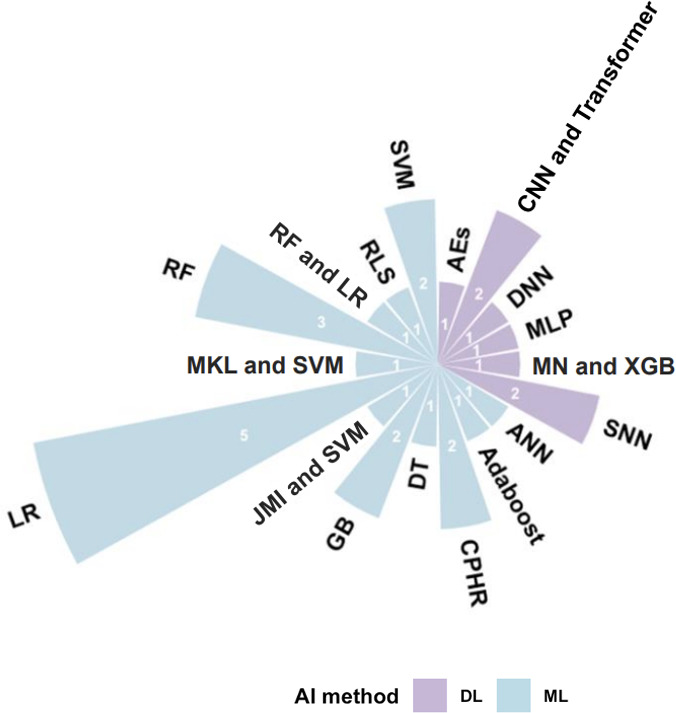
Distribution of artificial intelligence (AI) methodological approaches used in included magnetic resonance imaging (MRI)–based models. This figure displays the distribution of AI methodological approaches used in the included studies developing MRI-based AI models for predicting biochemical recurrence in individuals with pathologically confirmed prostate cancer. The chart categorizes approaches by machine learning (ML; light blue) and deep learning (DL; purple), with the size of each segment proportional to the number of studies using that specific method. AE: autoencoder; ANN: artificial neural network; CNN: convolutional neural network; CPHR: Cox proportional hazards regression; DNN: deep neural network; DT: decision trees; GB: gradient boosting; JMI: joint mutual information maximization; LR: logistic regression; MKL: multiple kernel learning; MLP: multilayer perceptron; MN: Medical Net; RF: random forest; RLS: regularized least squares; SNN: spiking neural network; SVM: support vector machine; XGB: Extreme Gradient Boosting; WAS: weighted average stacking.

**Figure 4.**
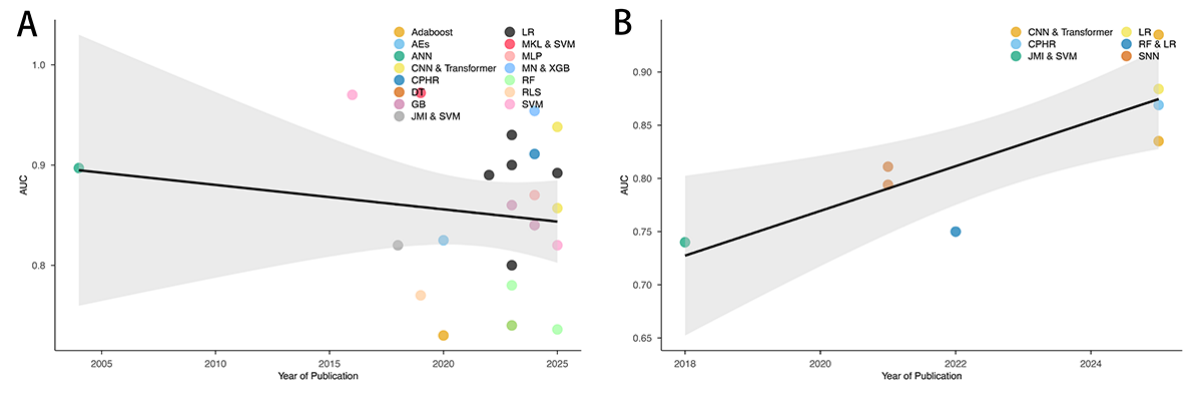
Bubble plots of area under the curve (AUC) by publication year for magnetic resonance imaging (MRI)–based artificial intelligence (AI) models predicting prostate cancer biochemical recurrence. This figure shows bubble plots depicting the association between publication year and AUC for MRI-based AI models predicting biochemical recurrence in individuals with pathologically confirmed prostate cancer, stratified by validation set: (A) internal validation sets and (B) external validation sets; bubble size corresponds to study sample size, with different colors denoting distinct AI algorithms. AUC, area under the curve; AE: autoencoder; ANN: artificial neural network; CNN: convolutional neural network; CPHR: Cox proportional hazards regression; DT: decision trees; GB: gradient boosting; JMI: joint mutual information maximization; LR: logistic regression; MKL: multiple kernel learning; MLP: multilayer perceptron; MN: Medical Net; RF: random forest; RLS: regularized least squares; SVM: support vector machine; XGB: Extreme Gradient Boosting.

**Figure 5.**
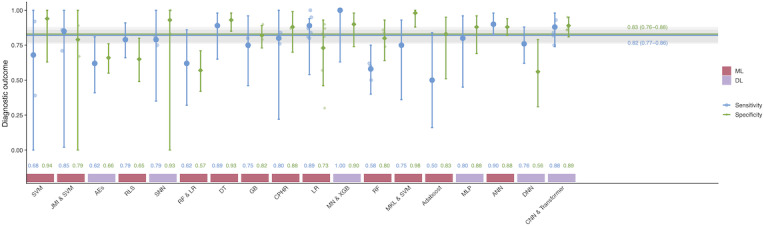
Point plots illustrating sensitivity and specificity of various artificial intelligence (AI) algorithms, with machine learning (ML) and deep learning (DL) distinguished. This figure presents the diagnostic performance of magnetic resonance imaging (MRI)–based AI models for predicting biochemical recurrence in individuals with pathologically confirmed prostate cancer, stratified by AI methodological approach. The plot displays sensitivity (blue circles) and specificity (green diamonds) for each AI method, with error bars representing 95% CIs. The bottom bar colors indicate whether the method is ML (red) or DL (purple). AE: autoencoder; ANN: artificial neural network; CNN: convolutional neural network; CPHR: Cox proportional hazards regression; DNN: deep neural network; DT: decision trees; GB: gradient boosting; LR: logistic regression; MLP: multilayer perceptron; MN: Medical Net; RF: random forest; RLS: regularized least squares; SNN: spiking neural network; SVM: support vector machine; XGB: Extreme Gradient Boosting.

As illustrated in Figure 3, among the total 29 datasets from 28 studies, the most commonly used traditional machine learning modeling algorithm was logistic regression (5/21, 24%), and the most frequently used deep learning modeling algorithms were spiking neural network (2/8, 25%) and convolutional neural network and transformer (2/8, 25%). In terms of the relationship between diagnostic performance and time, within the internal validation set, the AUC showed a slight downward trend from 2004 to 2025, with several algorithms (support vector machine [SVM], Medical Net and Extreme Gradient Boosting, and multiple kernel learning and SVM) achieving relatively high AUC values (Figure 4A). In the external validation set, the AUC showed an upward trend from 2018 to 2025 (Figure 4B). Regarding other diagnostic indicators, Medical Net and Extreme Gradient Boosting achieved the highest sensitivity, multiple kernel learning and SVM obtained the highest specificity (Figure 5). Notably, these comparisons are indirectly confounded by heterogeneous study designs (eg, different treatments and MRI protocols) and based on small sample sizes across individual studies.

### Internal and External Validation Sets

**Figure 6.**
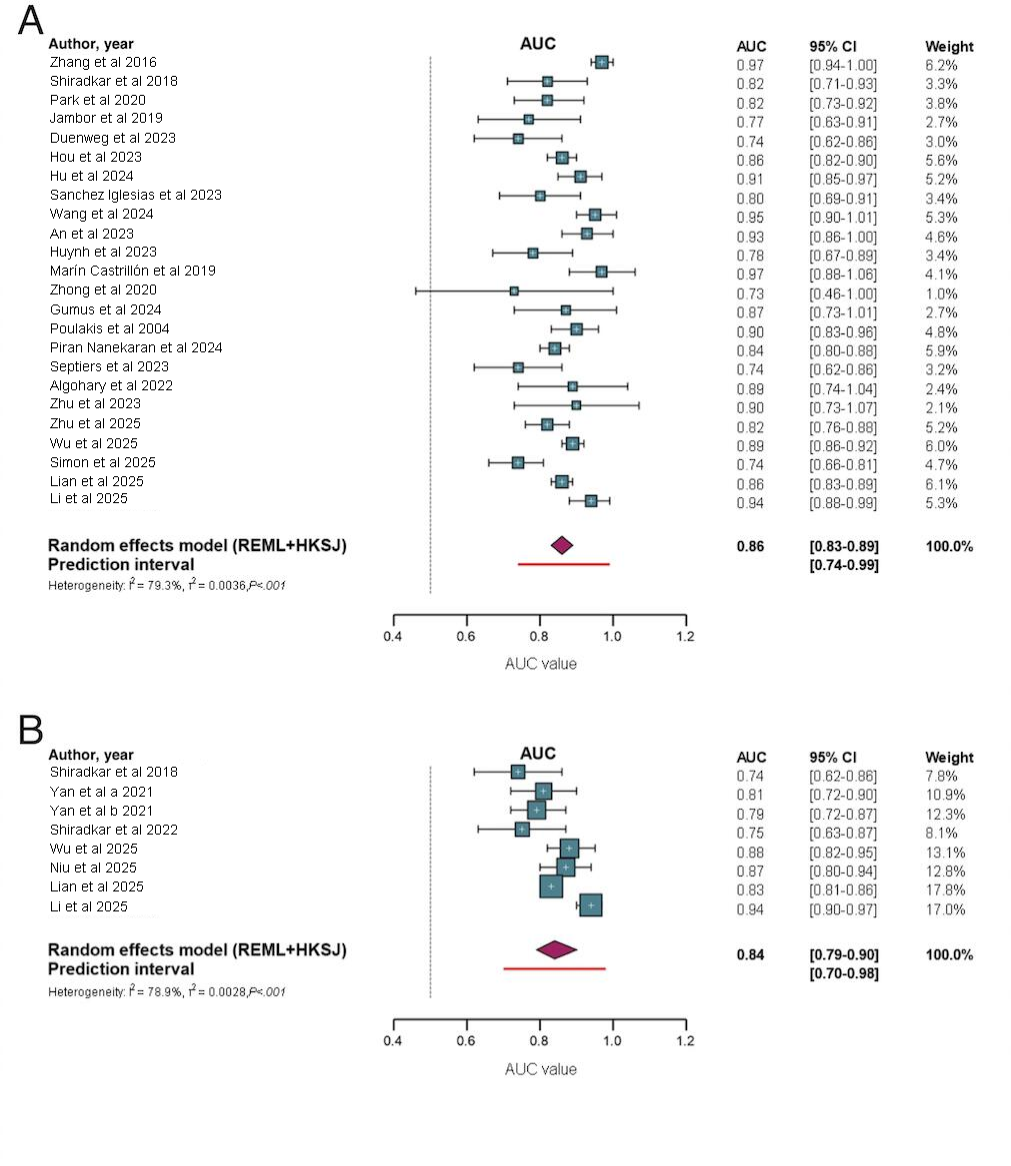
Forest plots of pooled diagnostic performance (area under the curve [AUC]) of magnetic resonance imaging (MRI)–based artificial intelligence (AI) models for predicting prostate cancer biochemical recurrence. This figure shows forest plots of the AUC of MRI-based AI models for predicting biochemical recurrence in individuals with pathologically confirmed prostate cancer, stratified by validation type. (A) Internal validation set results. (B) External validation set results. Each point represents an individual study, with horizontal lines indicating 95% CIs; pooled estimates and prediction intervals are shown at the bottom of each panel. AUC: area under the curve [11,18,22,27-51].

In the internal validation set, the sensitivity of the MRI-based AI model was 0.80 (95% CI 0.73‐0.86; PI 0.48‐0.99; τ^2^=0.02), the specificity was 0.83 (95% CI 0.77‐0.89; PI 0.49‐1.00; τ^2^=0.03), the DOR was 19.81 (95% CI 11.76‐33.38; PI 2.18‐179.81; τ^2^=1.07), and the AUC was 0.86 (95% CI 0.83‐0.89; PI 0.74‐0.99; τ^2^=0.004), as shown in Figures S1-S3A in Multimedia Appendix 1 and Figure 6A. In the external validation set, the sensitivity of the MRI-based AI model was 0.82 (95% CI 0.72‐0.91; PI 0.54‐0.99; τ^2^=0.01), the specificity was 0.83 (95% CI 0.71‐0.92; PI 0.49‐1.00; τ^2^=0.02), the DOR was 19.41 (95% CI 6.61‐56.95; PI 1.17‐321.94; τ^2^=1.19), and the AUC was 0.84 (95% CI 0.79‐0.90; PI 0.70‐0.98; τ^2^=0.002), as presented in Figures S1-S3B in Multimedia Appendix 1 and Figure 6B. No statistically significant differences were observed between the internal and external validation sets in terms of sensitivity (*P*=.73), specificity (*P*>.99), AUC (*P*=.53), or DOR (*P*=.98; Table S4 in Multimedia Appendix 1).

### Subgroup Analysis Based on Different Treatments

**Figure 7.**
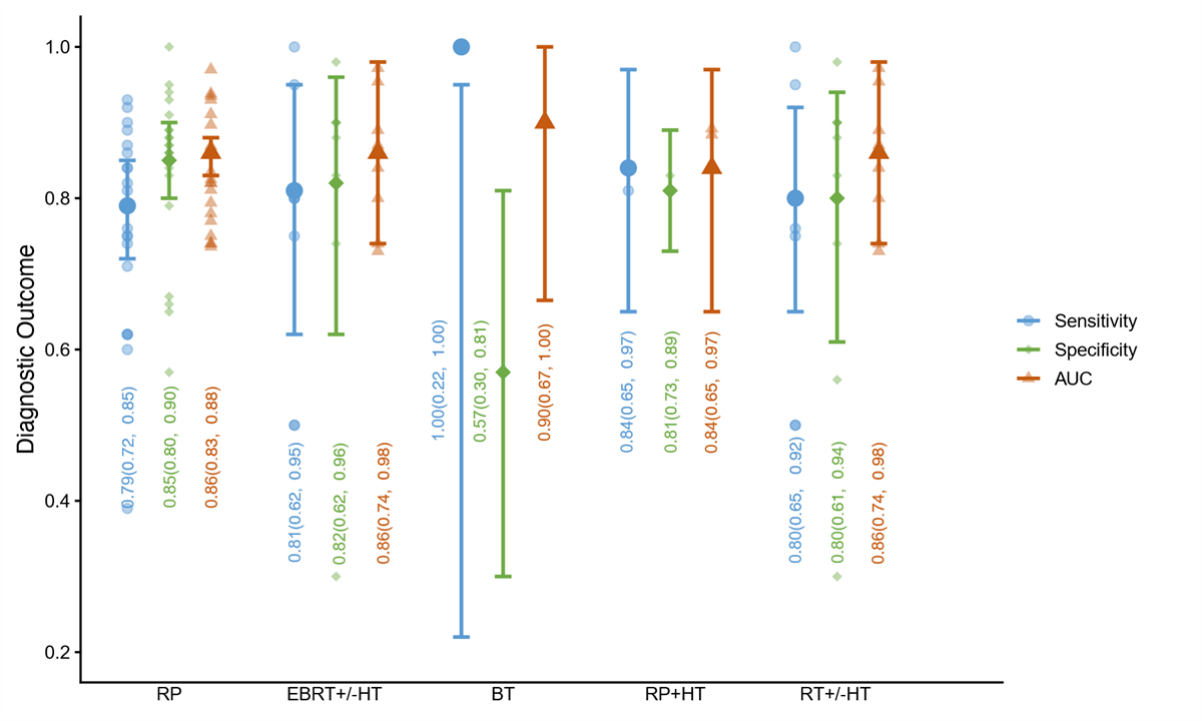
Point plots of sensitivity, specificity, and area under the curve (AUC) for magnetic resonance imaging (MRI)–based artificial intelligence (AI) across different prostate cancer treatments in predicting prostate cancer biochemical recurrence. It presents the diagnostic performance of MRI-based AI models for predicting biochemical recurrence in individuals with pathologically confirmed prostate cancer, stratified by primary treatment modality. The plot displays sensitivity (blue circles), specificity (green diamonds), and AUC (red triangles) for each treatment group, with error bars representing 95% CIs. The treatment modalities include radical prostatectomy (RP), external beam radiation therapy with or without hormone therapy (EBRT±HT), brachytherapy (BT), radical prostatectomy plus hormone therapy (RP+ HT), and radiation therapy with or without hormone therapy (RT±HT).

As shown in Figure 7, for patients who received RP, the sensitivity of the MRI-based AI model was 0.79 (95% CI 0.72‐0.85), the specificity was 0.85 (95% CI 0.80‐0.90), and the AUC was 0.86 (95% CI 0.83‐0.88). In patients treated with EBRT with or without HT, the sensitivity of the MRI-based AI model was 0.81 (95% CI 0.62‐0.95), the specificity was 0.82 (95% CI 0.62‐0.96), and the AUC was 0.86 (95% CI 0.74‐0.98). For patients undergoing brachytherapy, the sensitivity was 1.00 (95% CI 0.22‐1.00), the specificity was 0.57 (95% CI 0.30‐0.81), and the AUC was 0.90 (95% CI 0.67‐1.00). In patients treated with RP with HT, the sensitivity was 0.84 (95% CI 0.65‐0.97), the specificity was 0.81 (95% CI 0.73‐0.89), and the AUC was 0.84 (95% CI 0.65‐0.97). For patients receiving RT with or without HT, the sensitivity was 0.80 (95% CI 0.65‐0.92), the specificity was 0.80 (95% CI 0.61‐0.94), and the AUC was 0.86 (95% CI 0.74‐0.98). Notably, all treatment-stratified subgroup findings are hypothesis generating and should be interpreted cautiously, given the limited number of studies, small sample sizes, and wide CIs in specific treatment subgroups.

### Bivariate Box Plots and Meta-Regression

**Figure 8.**
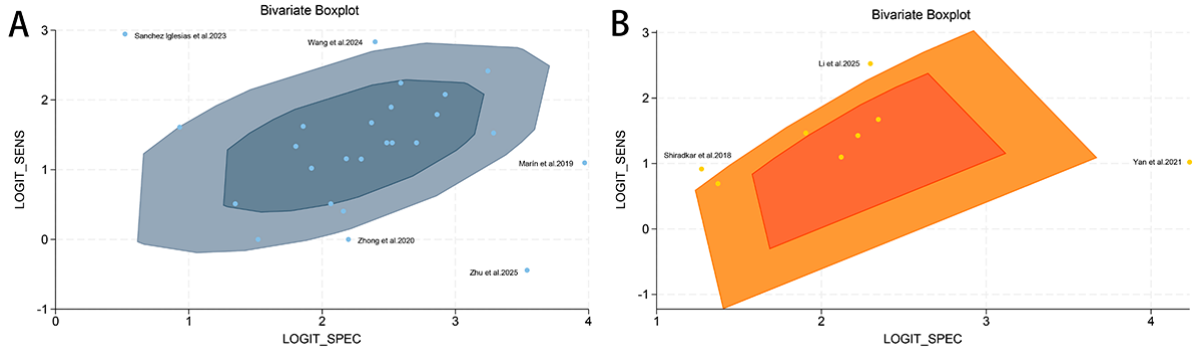
Bivariate boxplots of logit-transformed sensitivity (LOGIT_SENS) and specificity (LOGIT_SPEC) for magnetic resonance imaging (MRI)–based artificial intelligence models predicting biochemical recurrence in individuals with pathologically confirmed prostate cancer, stratified by validation type. (A) Results from internal validation sets [37,41,44,51]. (B) Results from external validation sets [39,46]. Each data point represents an individual study, with select studies labeled for reference. Points within the shaded regions represent studies whose diagnostic performance falls within the central distribution, while points outside the shaded regions represent outliers with performance outside this main cluster.

For the internal validation set, results from the bivariate box plots indicated that Sanchez Iglesias et al [37], Wang et al [41], Zhong et al [44], Zhu et al [51], and Marín Castrillón et al [29] might be the sources of heterogeneity (Figure 8A). For the external validation set, Shiradkar et al [39], Li et al [46], and Yan et al [22] might be the sources of heterogeneity (Figure 8B). Results of the Meta-regression analysis for the internal validation set revealed that several factors might be sources of heterogeneity, including AI method (machine learning vs deep learning, *P*=.03 for sensitivity and *P*=.03 for specificity), AI model (radiomic vs radiomic and clinical, *P*=.01 for specificity), time to acquire MRI image (pretreatment vs posttreatment, *P*<.001 for specificity), and treatment (RP vs RT with or without HT, *P*=.06 for sensitivity, *P*<.001 for specificity; Table 3).

**Table 3.** Subgroup analysis and meta-regression analysis of magnetic resonance imaging (MRI)–based artificial intelligence (AI) in internal validation sets for biochemical recurrence.

Subgroup	Studies, n	Sensitivity (95% CI)	Meta-regression *P* value	Specificity (95% CI)	Meta-regression *P* value
Analysis			.49		.81
Patient based	23	0.80 (0.73‐0.86)		0.84 (0.79‐0.90)	
Lesion based	2	0.79 (0.57‐1.00)		0.88 (0.74‐1.00)	
AI method			.03		.03
Machine learning	18	0.80 (0.73‐0.87)		0.85 (0.79‐0.91)	
Deep learning	7	0.79 (0.67‐0.90)		0.85 (0.75‐0.94)	
AI model			.12		.01
Radiomic	9	0.77 (0.65‐0.89)		0.85 (0.77‐0.94)	
Radiomic and clinical	16	0.80 (0.74‐0.87)		0.84 (0.78‐0.90)	
MRI sequence			.24		.06
bpMRI^a^	6	0.74 (0.60‐0.88)		0.86 (0.75‐0.96)	
mpMRI^b^	14	0.78 (0.70‐0.85)		0.83 (0.76‐0.91)	
Time to acquire MRI image			.60		<.001
Pretreatment	21	0.79 (0.72‐0.85)		0.86 (0.82‐0.91)	
Posttreatment	3	0.85 (0.71‐0.99)		0.57 (0.34‐0.80)	
Treatment			.06		<.001
RP^c^	15	0.79 (0.72‐0.86)		0.87 (0.81‐0.92)	
RT^d^±HT^e^	10	0.80 (0.69‐0.91)		0.79 (0.69‐0.90)	

a bpMRI: biparametric magnetic resonance imaging.

b mpMRI: multiparametric magnetic resonance imaging.

c RP: radical prostatectomy.

d RT: radiation therapy.

e HT: hormone therapy.

### Clinical Utility and Small-Study Effects

Using a median BCR positivity rate of 28% (IQR 18%-31%) as the prior probability, the Fagan nomogram showed a positive posttest probability of 66% and a negative posttest probability of 8% for the internal set, and 63% and 7% for the external set (Figure S4a-b in Multimedia Appendix 1). While statistically derived pooled estimates suggest potential clinical utility, the wide PIs and between-study variability indicate that real-world performance may differ substantially across clinical contexts. Deeks test showed no significant small-study effects in the internal set (*P*=.42), while the external set showed a nonsignificant trend toward small-study effects (*P*=.09), which should be interpreted with caution (Figure S5a-b in Multimedia Appendix 1).

## Discussion

Our meta-analysis demonstrates that MRI-based AI models showed broadly comparable diagnostic performance in internal and external validation for predicting BCR of PCa. This pattern aligns with established methodological challenges in previous research [1,4]. Overfitting risk in internal validation using institution-specific data splits [15,31] and covariate shift arising from variations in MRI protocols, scanners, and preprocessing may compromise model generalizability (eg, a decline in AUC from 0.74 to 0.60 across vendors) [18,20], and the reproducibility of radiomic features remains limited in the absence of rigorous harmonization procedures [22]. These factors may lead to overly optimistic internal performance estimates, highlighting the critical importance of external validation for the clinical translation of AI models [52].

Treatment modality appeared to contribute to variability in model performance. MRI-based AI showed broadly comparable performance between RP and RT with or without HT cohorts at the pooled level, with no meaningful differences in diagnostic performance [33,44]. Treatment-stratified estimates were heterogeneous and should be interpreted cautiously, given the wide CIs in several subgroups [53]. In radiotherapy-related cohorts, performance varied across EBRT with or without HT and brachytherapy subgroups, but these findings were accompanied by substantial uncertainty. This variability may reflect treatment-related domain shift and differences in tissue response and imaging appearance, and hormone therapy may further alter glandular morphology and cellularity, potentially affecting feature extraction and radiomics signatures (eg, fibrosis) [28,32]. In contrast, RT cohorts without concurrent HT may retain more distinct radiation-induced alterations as robust imaging features [31,44] and benefit from standardized posttreatment surveillance protocols that enhance feature reproducibility [31,37]. Brachytherapy patients, on the other hand, showed a pattern of higher sensitivity but lower specificity, likely related to inflammatory artifacts [45], further highlighting the treatment-specific impacts on model generalizability.

Interestingly, our meta-analysis demonstrates that radiomics and clinical models consistently achieve prominently higher sensitivity, yet moderately lower specificity, compared to radiomics-only models [54]. This observation was corroborated by multiple included studies: Poulakis et al [18] showed that combining MRI-derived radiomics with clinical variables (such as PSA and Gleason score) raised sensitivity from 0.81 to 0.89 but reduced specificity from 0.82 to 0.71; Jambor et al [34] and Hou et al [11] also reported that the integration of clinical risk factors and imaging features enhances the model’s ability to identify true BCR cases, but at the expense of increased FPs [11,34]. Mechanistically, this phenomenon can be attributed to the complementary information from multisource data fusion: clinical variables capture systemic and biological aspects of tumor aggressiveness not reflected in imaging, which increases detection of at-risk patients (higher sensitivity); however, many clinical high-risk markers (eg, elevated PSA and higher Gleason score) are nonspecific and may also be present in patients without recurrence, increasing the likelihood of misclassification and thus lowering specificity [34,35,45,55]. This trade-off is further supported by other included studies such as Park et al [35], Zhu et al [45], and Sun et al [56], all of which found that adding clinical variables to radiomics models improved recall but led to a rise in FP results. This trade-off between elevated sensitivity and reduced specificity highlights that optimizing the sensitivity-specificity balance is a core challenge for clinical AI model development [57]. The ideal balance is context dependent, varying with the clinical consequences of missed BCR (FNs) versus unnecessary interventions (FPs) [58]. Future model development must prioritize context-specific optimization of this balance to maximize practical clinical value [59].

In our meta-analysis, subgroup analysis based on the timing of MRI image acquisition revealed that models using pretreatment MRI exhibited improved predictive specificity for BCR compared to those using posttreatment MRI [60]. This finding is consistent with prior research, which suggests that pretreatment MRI provides more reliable information on tumor characteristics, as it is unaffected by posttreatment changes, such as fibrosis, necrosis, or tissue remodeling [61]. In contrast, posttreatment MRI may be influenced by these treatment-induced alterations, which can mimic tumor recurrence and confound image interpretation [62]. Such nontumor-related changes are more likely to appear on posttreatment scans, potentially leading to misinterpretation and FPs, thereby reducing specificity [63]. Therefore, careful consideration of MRI timing is critical for optimizing the clinical utility of MRI-based AI models for BCR prediction, particularly for enhancing specificity and minimizing FPs [64].

A recent systematic review by Liu et al [1] provided a valuable overview of AI applications for predicting BCR following RP, highlighting that MRI-based AI models achieved the highest median AUC-ROC (0.90) compared to models using pathological or clinicopathological variables alone. While Liu et al [65] offered important insights, their review focused specifically on postprostatectomy cohorts and did not perform meta-analytic synthesis or examine the influence of different treatment modalities. Our study builds upon this foundation by being the first to quantitatively synthesize the diagnostic performance of MRI-based AI models for BCR prediction across both RP and RT populations [11]. In addition, we directly evaluate the impact of treatment type on model accuracy.

Furthermore, a meta-analysis by Salimi et al [5] specifically pooled data from 24 studies of MRI-based radiomics models for BCR prediction, reporting pooled sensitivity, specificity, and AUC of 0.72, 0.78, and 0.75 for radiomics-only models, with clinical-radiomics models reaching an AUC of 0.88. While our pooled estimates align with Salimi et al, our meta-analysis introduces key methodological advances. Specifically, we are the first to stratify and compare model performance between internal and external validation cohorts. This novel analysis provides crucial quantitative evidence on model generalizability—essential for clinical translation [66]. Our findings show robust performance in internal validation and a slightly lower, but not statistically significant, AUC in external settings. This highlights the critical need for multicenter data harmonization and rigorous external testing [67]. In summary, these enhancements make our meta-analysis the first to comprehensively evaluate MRI-based AI for BCR prediction across diverse treatments and validation paradigms, offering new insights for future research and clinical use [11].

Our analysis identified substantial heterogeneity across studies that may affect the pooled diagnostic performance. Meta-regression suggested that AI method, AI model type, timing of MRI acquisition, and treatment modality were potential contributors to heterogeneity [68]. Several studies in both internal and external validation cohorts behaved as potential outliers and may have contributed to between-study variability. In addition to these identified factors, other unmeasured variables, such as differences in image preprocessing strategies and modeling approaches, may also have contributed to the observed heterogeneity [22]. These findings underscore the necessity of enhanced methodological standardization to improve model generalizability [20]. To address this heterogeneity, unified data protocols and reporting standards are imperative: uniform MRI acquisition and preprocessing protocols should be implemented to reduce equipment-related variability; diagnostic contingency tables (TP, FP, FN, or TN) should be explicitly reported to eliminate back-calculation and improve transparency; and consensus-based BCR definition guidelines should be adopted to reduce diagnostic-criterion heterogeneity across studies [69].

This meta-analysis has several notable limitations that may affect the interpretation and generalizability of its findings. First, most included studies were retrospective, with very few prospective studies included [27,30]. Retrospective designs inherently carry a higher risk of selection and information bias, particularly in the context of AI model training where unmeasured confounding and dataset imbalance may compromise predictive validity [70]. Second, many studies involved small sample sizes in either the training or validation cohorts [71]. Such limited datasets increase the risk of overfitting and reduce statistical robustness, potentially inflating diagnostic performance metrics [72]. Future studies should incorporate larger, balanced datasets with independent validation to improve model robustness [73]. Third, the definition of BCR was not uniform across all included studies. Although the AUA and Phoenix criteria were most commonly used, some studies used alternative or study-specific definitions, which may introduce inconsistency in diagnostic labeling. Although our subgroup analyses did not suggest meaningful performance differences between different reference standards, the lack of standardization remains a concern and highlights the need for consensus-based guidelines in future research [74]. Fourth, although the Deeks’ funnel plot did not indicate small-study effects, the limited number of included studies reduced the statistical power of this test, and the possibility of bias cannot be entirely excluded [75]. Fifth, selecting the best-performing model from each study may introduce optimistic bias. By focusing on top-performing models in each individual study, the performance metrics may be overly optimistic and not representative of real-world clinical outcomes [76]. In addition to overfitting, another significant limitation of the included AI models is their limited interpretability and transparency, which impedes clinical trust and regulatory acceptance [77]. Finally, and critically, due to lack of data, this study could not compare AI performance against MRI radiologists. This head-to-head comparison is indispensable for determining whether AI systems perform better than, equivalent to, or worse than human experts, serving as a key prerequisite for their clinical implementation [78]. Without this critical human benchmark, it remains unclear whether these MRI-based AI models offer genuine incremental clinical value or merely replicate the existing diagnostic capabilities of radiologists [79]. Prior systematic reviews have highlighted that while AI models sometimes report diagnostic accuracy comparable to or exceeding that of radiologists, these results often lack consistency when evaluated on independent datasets or in real-world settings [80]. As radiologist-level benchmarking is crucial for clinical adoption, future studies should directly compare AI tools against radiologists across different levels of expertise [81].

This meta-analysis innovatively quantifies and compares MRI-based AI model performance for BCR prediction between internal and external validation cohorts—a core clinical translation issue understudied in prior research, and our findings confirm these models exhibit promising diagnostic performance in both cohorts [82]. Distinct from previous reviews limited to single treatment cohorts or radiomics-only models, this study comprehensively evaluates AI performance across all major PCa treatment modalities and integrates machine learning and deep learning approaches, with subgroup findings revealing that AI models perform particularly well in patients who received EBRT alone [83]. These insights may inform personalized follow-up protocols based on treatment modality and risk stratification. In terms of clinical practicality, AI could augment radiologists’ ability to interpret complex MRI data, especially by extracting subtle, high-dimensional imaging features beyond the perceptual threshold of human observers. This capability may lead to earlier detection of recurrence risks and improved decision-making around salvage therapy [10]. Nevertheless, caution is warranted. PCa management involves multifactorial decision-making that integrates clinical context, patient preferences, comorbidities, and functional outcomes—areas where human judgment remains indispensable [80]. Therefore, AI should be positioned as a support system rather than a replacement for human expertise. Importantly, while algorithmic refinement and model optimization remain active areas of research, future gains in clinical applicability may depend more critically on improving the precision and consistency of imaging data itself [84]. Variability in image acquisition protocols—including differences in scanner hardware, field strength, pulse sequences, and operator technique—can significantly affect the quality of input data and, by extension, the reliability of AI outputs [85,86]. Enhancing the standardization of MRI protocols and ensuring high-fidelity imaging across institutions may serve as a more foundational step toward improving model robustness and generalizability [87]. Finally, the future of AI in PCa imaging will likely depend on its ability to synthesize multimodal data—including radiologic, genomics, and clinical biomarkers—into cohesive, interpretable frameworks [88]. Advances in federated learning and privacy-preserving technologies may also help overcome data-sharing barriers and facilitate collaborative model development across institutions [89,90]. Addressing these technical and regulatory challenges is essential for translating AI innovations into improved patient outcomes and more efficient PCa care pathways [91].

In conclusion, this meta-analysis contributes by quantitatively comparing the generalizability of MRI-based AI models through a synthesis of their performance in internal versus external validation for BCR prediction and further examines the influence of different treatment modalities on model performance relative to earlier reviews. Treatment-stratified analyses suggested heterogeneous performance across modalities, particularly within radiotherapy-related subgroups; however, these findings should be interpreted as hypothesis generating, given limited study numbers and wide CIs. Pretreatment MRI was identified to yield superior diagnostic specificity for BCR prediction, providing actionable guidance for future AI model development and clinical imaging workflow design. Collectively, these findings indicate that MRI-based AI models are promising investigational tools for PCa BCR prediction but are not yet ready for routine clinical use, due to the performance variation in external validation, the predominance of retrospective evidence, and unresolved methodological limitations. These tools may serve as adjunctive support for clinical decision-making in specific research settings, and their broader clinical translation requires large, prospective multicenter studies with harmonized imaging standards, rigorous external validation, and improved model interpretability.

## Supplementary material

10.2196/85360 Multimedia Appendix 1 Detailed methodology, search strategies, quality assessments, subgroup analyses, and sensitivity analyses.

10.2196/85360 Checklist 1 PRISMA-DTA checklist.

10.2196/85360 Checklist 2 PRISMA 2020 for abstracts checklist.

10.2196/85360 Checklist 3 PRISMA-S checklist.

10.2196/85360 Checklist 4 Compliance with the CHARMS framework.

## Abbreviations

AI: artificial intelligence
AUA: American Urological Association
AUC: area under the curve
BCR: biochemical recurrence
CHARMS: Checklist for Critical Appraisal and Data Extraction for Systematic Reviews of Prediction Modelling Studies
DOR: diagnostic odds ratio
EBRT: external beam radiation therapy
FN: false negative
FP: false positive
GAIDeT: Generative AI Delegation Taxonomy
Gen-AI: generative artificial intelligence
HT: hormone therapy
MeSH: Medical Subject Headings
MRI: magnetic resonance imaging
PCa: prostate cancer
PI: prediction interval
PRISMA: Preferred Reporting Items for Systematic Reviews and Meta-Analyses
PRISMA-DTA: Preferred Reporting Items for Systematic Reviews and Meta-Analyses of Diagnostic Test Accuracy
PSA: prostate-specific antigen
ROC: receiver operating characteristic
RP: radical prostatectomy
RT: radiation therapy
SVM: support vector machine
TN: true negative
TP: true positive
