# Supplementary material for: Magnetic Resonance Imaging–Based Artificial Intelligence in Predicting Prostate Cancer Biochemical Recurrence: Systematic Review and Meta-Analysis
Source: J Med Internet Res. 2026 Jul 7;28:e85360. doi: 10.2196/85360 (PMC13340575; doi:10.2196/85360)

**Table of Contents**

[Table S1 Search strategy in PubMed, Embase, Web of Science, and Cochrane Library. 10](#_Toc228479299)

[Table S2. Risk of bias assessment (PROBAST+AI) model development based on seven domains. 13](#_Toc228479300)

[Table S3. Risk of bias assessment (PROBAST+AI) model evaluation based on seven domains. 16](#_Toc228479301)

[Table S4. Z-test for subgroup differences in sensitivity, specificity, and AUC between internal and external validation sets. 19](#_Toc228479302)

[Figure S1. Forest plots of sensitivity: (A) internal validation sets and (B) external validation sets. 20](#_Toc228479303)

[Figure S2. Forest plots of specificity: (A) internal validation sets and (B) external validation sets. 21](#_Toc228479304)

[Figure S3. Forest Plots of Diagnostic Odds Ratio (DOR): (A) internal validation sets and (B) external validation sets. 22](#_Toc228479305)

[Figure S4. Fagan plots for assessing clinical applicability: (A) internal validation sets and (B) external validation sets. 23](#_Toc228479306)

[Figure S5. Deeks’ Funnel plot asymmetry test for evaluating publication bias: (A) internal validation sets and (B) external validation sets. 24](#_Toc228479307)

### Table S1. Search strategy in PubMed, Embase, Web of Science, and Cochrane Library.

| Database | Search strategy |
| --- | --- |
| PubMed | #1 "Prostatic Neoplasms"[Mesh] OR "prostate cancer"[Title/Abstract] OR "prostatic cancer"[Title/Abstract] OR "prostate tumor*"[Title/Abstract] OR "prostate tumour*"[Title/Abstract] OR "prostatic neoplasm*"[Title/Abstract] OR "prostate carcinoma*"[Title/Abstract] OR "prostatic adenocarcinoma"[Title/Abstract] OR "prostate adenocarcinoma"[Title/Abstract] OR "PCa"[Title/Abstract]  #2 "Artificial Intelligence"[Mesh] OR "Machine Learning"[Mesh] OR "Deep Learning"[Mesh] OR "Neural Networks, Computer"[Mesh] OR "Radiometry"[Mesh] OR "artificial intelligence"[Title/Abstract] OR "machine learning"[Title/Abstract] OR "deep learning"[Title/Abstract] OR  "radiomic*"[Title/Abstract] OR "radiogenomic*"[Title/Abstract] OR "neural network*"[Title/Abstract] OR "support vector machine*"[Title/Abstract] OR "random forest*"[Title/Abstract] OR "convolutional neural network*"[Title/Abstract] OR "CNN"[Title/Abstract] OR "transformer"[Title/Abstract] OR "XGBoost"[Title/Abstract] OR "gradient boost*"[Title/Abstract] OR "logistic regression"[Title/Abstract] OR "decision tree*"[Title/Abstract] OR "ensemble learning"[Title/Abstract] OR "federated learning"[Title/Abstract] OR "transfer learning"[Title/Abstract] OR "feature extraction"[Title/Abstract] OR "image segmentation"[Title/Abstract] OR "computer-aided diagnos*"[Title/Abstract] OR "CAD"[Title/Abstract] OR "automated detection"[Title/Abstract] OR "prediction model*"[Title/Abstract] OR "prognostic model*"[Title/Abstract]  #3 "Magnetic Resonance Imaging"[Mesh] OR "magnetic resonance imaging"[Title/Abstract] OR "MRI"[Title/Abstract] OR "mpMRI"[Title/Abstract] OR "multiparametric MRI"[Title/Abstract] OR "MR imaging"[Title/Abstract] OR "diffusion weighted imaging"[Title/Abstract] OR "DWI"[Title/Abstract] OR "dynamic contrast enhanced"[Title/Abstract] OR "DCE"[Title/Abstract] OR "apparent diffusion coefficient"[Title/Abstract] OR "ADC"[Title/Abstract] OR "T2 weighted"[Title/Abstract] OR "T2W"[Title/Abstract] OR "MR spectroscop*"[Title/Abstract] OR "prostate imaging"[Title/Abstract]  #4 "Neoplasm Recurrence, Local"[Mesh] OR "Recurrence"[Mesh] OR "biochemical recurrence"[Title/Abstract] OR "BCR"[Title/Abstract] OR "biochemical relapse"[Title/Abstract] OR "PSA recurrence"[Title/Abstract] OR "prostate-specific antigen recurrence"[Title/Abstract] OR "PSA failure"[Title/Abstract] OR "PSA progression"[Title/Abstract] OR "biochemical failure"[Title/Abstract] OR "treatment failure"[Title/Abstract] OR "disease recurrence"[Title/Abstract] OR "disease relapse"[Title/Abstract] OR "oncologic outcome*"[Title/Abstract] OR "cancer recurrence"[Title/Abstract] OR "recurrence-free survival"[Title/Abstract] OR "biochemical progression"[Title/Abstract]  #5 #1 AND #2 AND #3 AND #4 |
| Embase | #1 'prostate tumor'/exp OR 'prostate cancer':ti,ab,kw OR 'prostatic cancer':ti,ab,kw OR 'prostate tumor*':ti,ab,kw OR 'prostate tumour*':ti,ab,kw OR 'prostatic neoplasm*':ti,ab,kw OR 'prostate carcinoma*':ti,ab,kw OR 'prostatic adenocarcinoma':ti,ab,kw OR 'prostate adenocarcinoma':ti,ab,kw OR 'PCa':ti,ab,kw  #2 'artificial intelligence'/exp OR 'machine learning'/exp OR 'deep learning'/exp OR 'radiomics'/exp OR 'artificial neural network'/exp OR  'artificial intelligence':ti,ab,kw OR 'machine learning':ti,ab,kw OR 'deep learning':ti,ab,kw OR 'radiomic*':ti,ab,kw OR 'radiogenomic*':ti,ab,kw OR 'neural network*':ti,ab,kw OR 'support vector machine*':ti,ab,kw OR 'random forest*':ti,ab,kw OR 'convolutional neural network*':ti,ab,kw OR 'CNN':ti,ab,kw OR 'transformer':ti,ab,kw OR 'XGBoost':ti,ab,kw OR 'gradient boost*':ti,ab,kw OR 'logistic regression':ti,ab,kw OR 'decision tree*':ti,ab,kw OR 'ensemble learning':ti,ab,kw OR 'transfer learning':ti,ab,kw OR 'computer aided diagnos*':ti,ab,kw OR 'prediction model*':ti,ab,kw OR 'prognostic model*':ti,ab,kw OR 'automated detection':ti,ab,kw  #3 'nuclear magnetic resonance imaging'/exp OR 'magnetic resonance imaging':ti,ab,kw OR 'MRI':ti,ab,kw OR 'mpMRI':ti,ab,kw OR 'multiparametric MRI':ti,ab,kw OR 'MR imaging':ti,ab,kw OR 'diffusion weighted imaging':ti,ab,kw OR 'DWI':ti,ab,kw OR 'dynamic contrast enhanced':ti,ab,kw OR 'DCE':ti,ab,kw OR 'apparent diffusion coefficient':ti,ab,kw OR 'ADC':ti,ab,kw OR 'T2 weighted':ti,ab,kw OR 'T2W':ti,ab,kw OR 'prostate imaging':ti,ab,kw  #4 'biochemical recurrence'/exp OR 'recurrent disease'/exp OR 'biochemical recurrence':ti,ab,kw OR 'BCR':ti,ab,kw OR 'biochemical relapse':ti,ab,kw OR 'PSA recurrence':ti,ab,kw OR 'prostate-specific antigen recurrence':ti,ab,kw OR 'PSA failure':ti,ab,kw OR  'PSA progression':ti,ab,kw OR 'biochemical failure':ti,ab,kw OR 'treatment failure':ti,ab,kw OR 'disease recurrence':ti,ab,kw OR 'recurrence-free survival':ti,ab,kw OR 'biochemical progression':ti,ab,kw OR 'oncologic outcome*':ti,ab,kw  #5 #1 AND #2 AND #3 AND #4 |
| Web of Science | #1 TS=("prostate cancer" OR "prostatic cancer" OR "prostate tumor*" OR "prostate tumour*" OR "prostatic neoplasm*" OR "prostate carcinoma*" OR "prostatic adenocarcinoma" OR "prostate adenocarcinoma" OR "PCa")  #2 TS=("artificial intelligence" OR "machine learning" OR "deep learning" OR "radiomic*" OR "radiogenomic*" OR "neural network*" OR "support vector machine*" OR "random forest*" OR "convolutional neural network*" OR "CNN" OR "transformer" OR "XGBoost" OR "gradient boost*" OR "logistic regression" OR "decision tree*" OR "ensemble learning" OR "transfer learning" OR "computer-aided diagnos*" OR "prediction model*" OR "prognostic model*" OR "automated detection")  #3 TS=("magnetic resonance imaging" OR "MRI" OR "mpMRI" OR "multiparametric MRI" OR "MR imaging" OR "diffusion weighted imaging" OR "DWI" OR "dynamic contrast enhanced" OR "DCE" OR "apparent diffusion coefficient" OR "ADC" OR "T2 weighted" OR  "prostate imaging")  #4 TS=("biochemical recurrence" OR "BCR" OR "biochemical relapse" OR "PSA recurrence" OR "prostate-specific antigen recurrence" OR "PSA failure" OR "PSA progression" OR "biochemical failure" OR "treatment failure" OR "disease recurrence" OR "recurrence-free survival" OR "biochemical progression" OR "oncologic outcome*")  #5 #1 AND #2 AND #3 AND #4 |
| Cochrane Library | #1 ([mh "Prostatic Neoplasms"] OR ("prostate cancer" OR "prostatic cancer" OR "prostate tumor" OR "prostate tumors" OR "prostate tumour" OR "prostate tumours" OR "prostatic neoplasm" OR "prostatic neoplasms" OR "prostate carcinoma" OR "prostate carcinomas" OR "prostatic adenocarcinoma" OR "prostate adenocarcinoma" OR "PCa"):ti,ab,kw)  #2 ([mh "Artificial Intelligence"] OR [mh "Machine Learning"] OR [mh "Deep Learning"] OR [mh "Neural Networks, Computer"] OR ("artificial intelligence" OR "machine learning" OR "deep learning" OR "radiomics" OR "radiomic" OR "radiogenomics" OR "radiogenomic" OR "neural network" OR "neural networks" OR "support vector machine" OR "support vector machines" OR "SVM" OR "random forest" OR "random forests" OR "convolutional neural network" OR "convolutional neural networks" OR "CNN" OR "ResNet" OR "ResNets" OR "transformer" OR "transformers" OR "XGBoost" OR "LightGBM" OR "gradient boosting" OR "decision tree" OR "decision trees" OR "ensemble learning" OR "transfer learning" OR "logistic regression" OR "naive bayes" OR "naive bayesian" OR "multilayer perceptron" OR  "multilayer perceptrons" OR "adaboost" OR "bayesian network" OR "bayesian networks" OR "k-nearest neighbor" OR "k-nearest neighbors" OR "nomogram" OR "nomograms" OR "prediction model" OR "prediction models" OR "predictive model" OR "predictive models" OR  "prognostic model" OR "prognostic models" OR "diagnostic model" OR "diagnostic models" OR "risk model" OR "risk models" OR "risk score" OR "risk scores" OR "scoring system" OR "scoring systems" OR "prediction tool" OR "prediction tools" OR "computer-aided diagnosis" OR "computer-aided detection" OR "automated detection"):ti,ab,kw)  #3 ([mh "Magnetic Resonance Imaging"] OR ("magnetic resonance imaging" OR "MRI" OR "mpMRI" OR "multiparametric MRI" OR "MR imaging" OR "diffusion weighted imaging" OR "DWI" OR "dynamic contrast enhanced" OR "DCE" OR "apparent diffusion coefficient" OR "ADC" OR "T2 weighted" OR "T2W" OR "prostate imaging"):ti,ab,kw)  #4 ([mh "Neoplasm Recurrence, Local"] OR [mh "Recurrence"] OR ("biochemical recurrence" OR "biochemical recurrences" OR "BCR" OR  "biochemical relapse" OR "biochemical relapses" OR "PSA recurrence" OR "prostate-specific antigen recurrence" OR "PSA failure" OR "PSA progression" OR "biochemical failure" OR "biochemical failures" OR "treatment failure" OR "treatment failures" OR "disease recurrence" OR "disease recurrences" OR "recurrence-free survival" OR "biochemical progression" OR "oncologic outcome" OR "oncologic outcomes"):ti,ab,kw)  #5 #1 AND #2 AND #3 AND #4 |

### Table S2. Risk of bias assessment (PROBAST+AI) model development based on seven domains.

| Author, year | | Quality | | | | Applicability concerns | | | Overall judgement | |
| --- | --- | --- | --- | --- | --- | --- | --- | --- | --- | --- |
|  |  | Participants and data sources ^a^ | Predictors ^b^ | Outcome ^c^ | Analysis ^d^ | Participants and data sources ^e^ | Predictors ^f^ | Outcome ^g^ | Quality ^h^ | Applicability concerns ^i^ |
| Zhang et al. | 2016 | L | L | L | L | L | L | L | L | L |
| Shiradkar et al. | 2018 | L | H | L | H | L | L | L | H | L |
| Park et al. | 2020 | L | L | U | U | L | L | L | U | L |
| Jambor et al. | 2019 | L | L | U | H | L | L | L | H | L |
| Yan et al. | 2021 | L | U | U | L | L | L | L | U | L |
| Shiradkar et al. | 2022 | L | U | L | H | L | L | L | H | L |
| Duenweg et al. | 2023 | L | U | L | L | L | L | L | U | L |
| Hou et al. | 2023 | L | L | L | L | L | L | L | L | L |
| Hu et al. | 2021 | L | U | L | L | L | L | L | U | L |
| Sanchez Iglesias et al. | 2023 | L | U | L | U | L | L | L | U | L |
| Wang et al. | 2021 | L | U | U | H | L | L | L | H | L |
| Hu et al. | 2024 | L | U | U | L | L | L | L | U | L |
| An et al. | 2023 | L | L | U | L | L | L | L | U | L |
| Huynh et al. | 2023 | L | L | U | U | L | L | L | U | L |
| Marín et al. | 2019 | L | U | U | H | L | L | L | H | L |
| ​Zhong et al. | 2020 | L | U | U | H | L | L | L | H | L |
| Gumus et al. | 2024 | L | L | U | H | L | L | L | H | L |
| Poulakis et al. | 2004 | L | U | U | U | L | L | L | U | L |
| Yilmaz et al. | 2023 | L | L | L | H | L | L | L | H | L |
| Nanekaran et al. | 2024 | L | U | U | L | L | L | L | U | L |
| Septiers et al. | 2023 | L | U | U | U | L | L | L | U | L |
| Algohary et al. | 2022 | L | U | U | H | L | L | L | H | L |
| ​Zhu et al. | 2023 | L | U | U | H | L | L | L | H | L |
| Zhu et al. | 2025 | L | U | U | U | L | L | L | U | L |
| Wu et al. | 2025 | L | U | U | U | L | L | L | U | L |
| Simon et al. | 2025 | L | U | U | L | L | L | L | U | L |
| Niu et al. | 2025 | L | U | U | L | L | L | L | U | L |
| Lian et al. | 2025 | L | U | U | L | L | L | L | U | L |
| Li et al. | 2025 | L | U | U | L | L | L | L | U | L |

**Abbreviation:** PROBAST+AI, Prediction model Risk of Bias Assessment Tool + AI, L low; H high; U unclear.

**Footnote:** Signaling questions are rated as "yes" (Y), "probably yes" (PY), "probably no" (PN), "no" (N), "no information" (NI), and in some cases "not applicable" (NA). All signaling questions are phrased in such a way that "yes" or "probably yes" indicates a low risk of bias. Any signaling questions rated as "no" or "probably no" indicate a potential high risk of bias in that domain. If there are no "no" or "probably no" ratings, but "no information" (NI) is present, the risk of bias in that domain is classified as unclear.

a. Participants and data sources

1.1 Were appropriate data sources used?

1.2 Was an appropriate study design used?

1.3 Did the in- and exclusions of study participants result in a representative dataset?

b. Predictors

2.1 Were predictors defined and assessed in a similar way for all participants?

2.2 Was any pre-processing of predictors similar for all participants?

2.3 Were predictor assessments made without knowledge of outcome data?

2.4 Were the predictors included in the model available at the time the model was intended to be used?

c. Outcome

3.1 Were outcomes defined and assessed appropriately?

3.2 Were outcomes defined and assessed in a similar way for all participants?

3.3 Were outcome assessments made without use or knowledge of predictor data?

3.4 Was the time interval between predictor assessment and outcome assessment appropriate?

d. Analysis

4.1 Was there evidence that the sample size was reasonable?

4.2 Were continuous and categorical predictors handled appropriately?

4.3 Were participants with missing or censored data handled appropriately in the analysis?

4.4 If methods to address class imbalance were used, was the model or the model predictions recalibrated?

4.5 Were methods used to address potential model overfitting?

e. Participants and data sources

Concern that the (data of the) included participants do not match the review question or the assessor’s intended use of the prediction model.

f. Predictors

Concern that the definition, pre-processing, assessment, or timing of assessment of the predictors in the model do not match the review question or the assessor’s intended use.

g. Outcome

Concern that the outcome, its definition, assessment, or timing of assessment do not match the review question or the assessor’s intended use.

h. Quality

Low risk: If all four domains were rated low concern regarding quality.

High risk: If at least one domain was rated high concern regarding quality .

Unclear: If at least one domain was rated unclear concern regarding quality and no domains were rated high concern.

i. Applicability concerns

Low risk: If all three domains were rated low concern for applicability.

High risk: If at least one domain was rated high concern for applicability.

Unclear: If at least one domain was rated unclear concern for applicability and no domains were rated high concern.

### Table S3. Risk of bias assessment (PROBAST+AI) model evaluation based on seven domains.

| Author, year | | Risk of bias | | | | Applicability concerns | | | Overall judgement | |
| --- | --- | --- | --- | --- | --- | --- | --- | --- | --- | --- |
|  |  | Participants and data sources ^a^ | Predictors ^b^ | Outcome ^c^ | Analysis ^d^ | Participants and data sources ^e^ | Predictors ^f^ | Outcome ^g^ | Risk of bias ^h^ | Applicability concerns ^i^ |
| Zhang et al. | 2016 | L | L | L | L | L | L | L | L | L |
| Shiradkar et al. | 2018 | L | U | U | H | L | L | L | H | L |
| Park et al. | 2020 | L | L | U | U | L | L | L | U | L |
| Jambor et al. | 2019 | L | L | U | H | L | L | L | H | L |
| Yan et al. | 2021 | L | U | U | L | L | L | L | U | L |
| Shiradkar et al. | 2022 | L | U | L | H | L | L | L | H | L |
| Duenweg et al. | 2023 | L | U | L | L | L | L | L | U | L |
| Hou et al. | 2023 | L | L | L | L | L | L | L | L | L |
| Hu et al. | 2021 | L | U | L | L | L | L | L | U | L |
| Sanchez Iglesias et al. | 2023 | L | U | L | H | L | L | L | H | L |
| Wang et al. | 2021 | L | U | U | H | L | L | L | H | L |
| Hu et al. | 2024 | L | U | U | L | L | L | L | U | L |
| An et al. | 2023 | L | L | U | L | L | L | L | U | L |
| Huynh et al. | 2023 | L | L | U | U | L | L | L | U | L |
| Marín et al. | 2019 | L | U | U | H | L | L | L | H | L |
| ​Zhong et al. | 2020 | L | U | U | H | L | L | L | H | L |
| Gumus et al. | 2024 | L | L | U | H | L | L | L | H | L |
| Poulakis et al. | 2004 | L | U | U | U | L | L | L | U | L |
| Yilmaz et al. | 2023 | L | L | L | H | L | L | L | H | L |
| Nanekaran et al. | 2024 | L | U | U | H | L | L | L | H | L |
| Septiers et al. | 2023 | L | U | U | H | L | L | L | H | L |
| Algohary et al. | 2022 | L | U | U | H | L | L | L | H | L |
| ​Zhu et al. | 2023 | L | U | U | H | L | L | L | H | L |
| Zhu et al. | 2025 | L | U | L | U | L | L | L | U | L |
| Wu et al. | 2025 | L | U | L | U | L | L | L | U | L |
| Simon et al. | 2025 | L | U | L | L | L | L | L | U | L |
| Niu et al. | 2025 | L | U | L | L | L | L | L | U | L |
| Lian et al. | 2025 | L | U | L | L | L | L | L | U | L |
| Li et al. | 2025 | L | U | L | L | L | L | L | U | L |

**Abbreviation:** PROBAST+AI, Prediction model Risk of Bias Assessment Tool + AI, L low; H high; U unclear.

**Footnote:** Signaling questions are rated as "yes" (Y), "probably yes" (PY), "probably no" (PN), "no" (N), "no information" (NI), and in some cases "not applicable" (NA). All signaling questions are phrased in such a way that "yes" or "probably yes" indicates a low risk of bias. Any signaling questions rated as "no" or "probably no" indicate a potential high risk of bias in that domain. If there are no "no" or "probably no" ratings, but "no information" (NI) is present, the risk of bias in that domain is classified as unclear.

a. Participants and data sources

1.1 Were appropriate data sources used?

1.2 Was an appropriate study design used?

1.3 Did the in- and exclusions of study participants result in a representative dataset?

b. Predictors

2.1 Were predictors defined and assessed in a similar way for all participants?

2.2 Was any pre-processing of predictors similar for all participants?

2.3 Were predictor assessments made without knowledge of outcome data?

2.4 Were the predictors included in the model available at the time the model was intended to be used?

c. Outcome

3.1 Were outcomes defined and assessed appropriately?

3.2 Were outcomes defined and assessed in a similar way for all participants?

3.3 Were outcome assessments made without use or knowledge of predictor data?

3.4 Was the time interval between predictor assessment and outcome assessment appropriate?

d. Analysis

4.1 Was model evaluation based on only apparent performance avoided?

4.2 Was there evidence that the sample size was reasonable?

4.3 Were participants with missing or censored data handled appropriately in the analysis?

4.4 If methods to address class imbalance were used, was the evaluation done in a dataset without imbalance correction?

4.5 If data splitting was done to create training and test datasets, was there evidence that data leakage was avoided?

4.6 If resampling methods were used to evaluate model performance, were all model development steps replicated in the resampling process?

4.7 Was the predictive performance of the model evaluated appropriately, e.g., calibration, discrimination, and net benefit?

e. Participants and data sources

Concern that the (data of the) included participants do not match the review question or the assessor’s intended use of the prediction model.

f. Predictors

Concern that the definition, pre-processing, assessment, or timing of assessment of the predictors in the model do not match the review question or the assessor’s intended use.

g. Outcome

Concern that the outcome, its definition, assessment, or timing of assessment do not match the review question or the assessor’s intended use.

h. Risk of bias

Low risk: If all four domains were rated low risk of bias.

High risk: If at least one domain was rated high risk of bias.

Unclear: If at least one domain was rated unclear risk of bias and no domains were rated high risk of bias.

i. Applicability concerns

Low risk: If all three domains were rated low concern for applicability.

High risk: If at least one domain was rated high concern for applicability.

Unclear: If at least one domain was rated unclear concern for applicability and no domains were rated high concern.

### Table S4. Z-test for subgroup differences in sensitivity, specificity, and AUC between internal and external validation sets.

| Subgroup | Contingency tables, n | Sensitivity(95%CI) | Subgroup difference ^a^ | Specificity(95%CI) | Subgroup difference ^a^ | AUC(95%CI) | Subgroup difference ^a^ | DOR(95%CI) | Subgroup difference ^a^ |
| --- | --- | --- | --- | --- | --- | --- | --- | --- | --- |
|  |  |  | Z= 0.34, *P* = 0.73 |  | Z= 0.00, *P* = 1.00 |  | Z= 0.63, *P* = 0.53 |  | Z= 0.03, *P* = 0.98 |
| Internal validation sets | 25^b^ | 0.80 (0.73 - 0.86) |  | 0.83 (0.77 - 0.89) |  | 0.86 (0.83 - 0.89) |  | 19.81 (11.76 - 33.38) |  |
| External validation sets | 8 | 0.82 (0.72 - 0.91) |  | 0.83 (0.71 - 0.92) |  | 0.84 (0.79 - 0.90) |  | 19.41 (6.61 – 56.95) |  |

AUC area under the curve; DOR diagnostic odds ratio**.**

**^a^** Based on two-sided Z-test.

^b^ AUC from 24 articles is obtained in internal validation sets.

### Figure S1. Forest plots of sensitivity: (A) internal validation sets and (B) external validation sets.


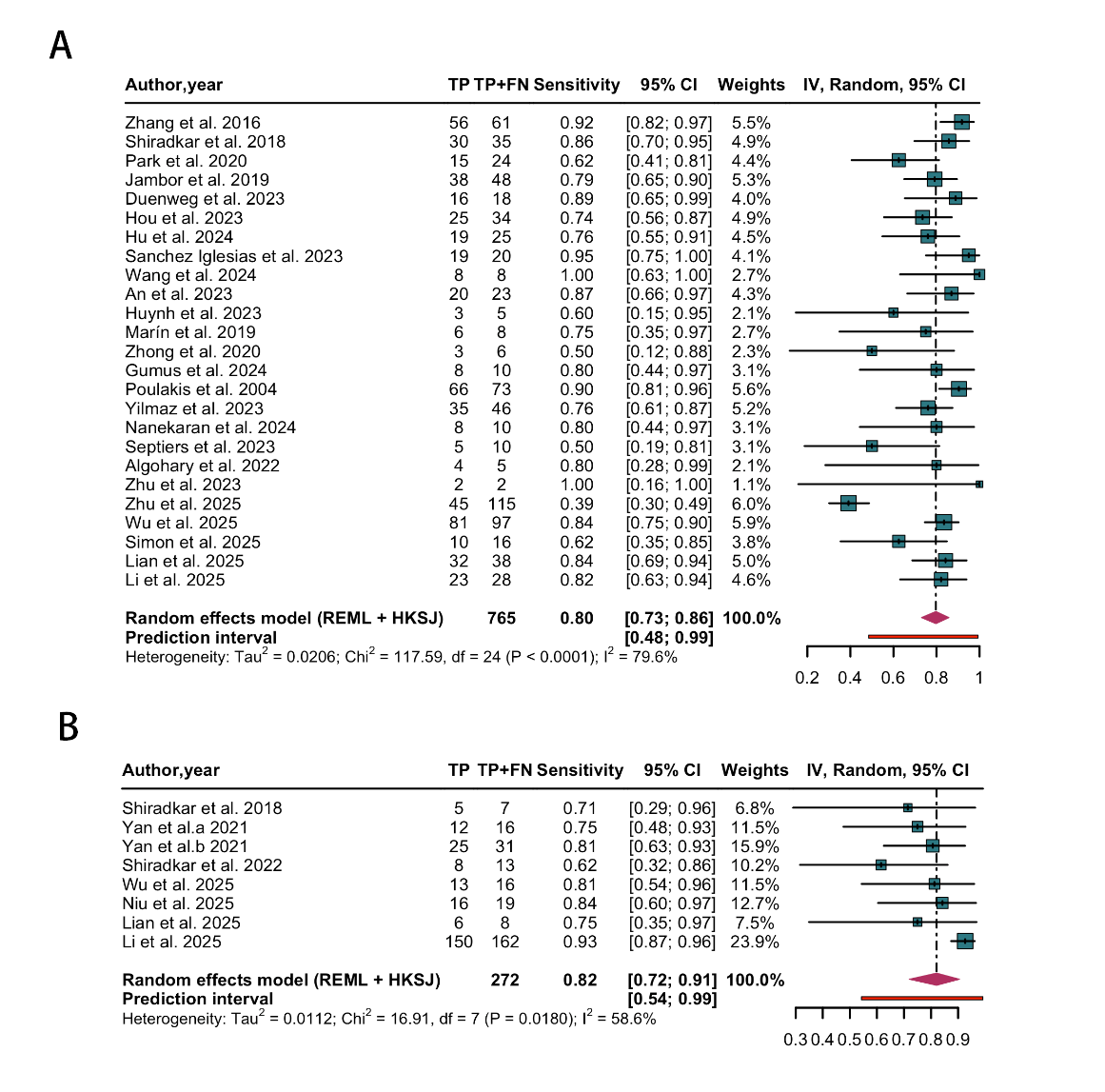


### Figure S2. Forest plots of specificity: (A) internal validation sets and (B) external validation sets.


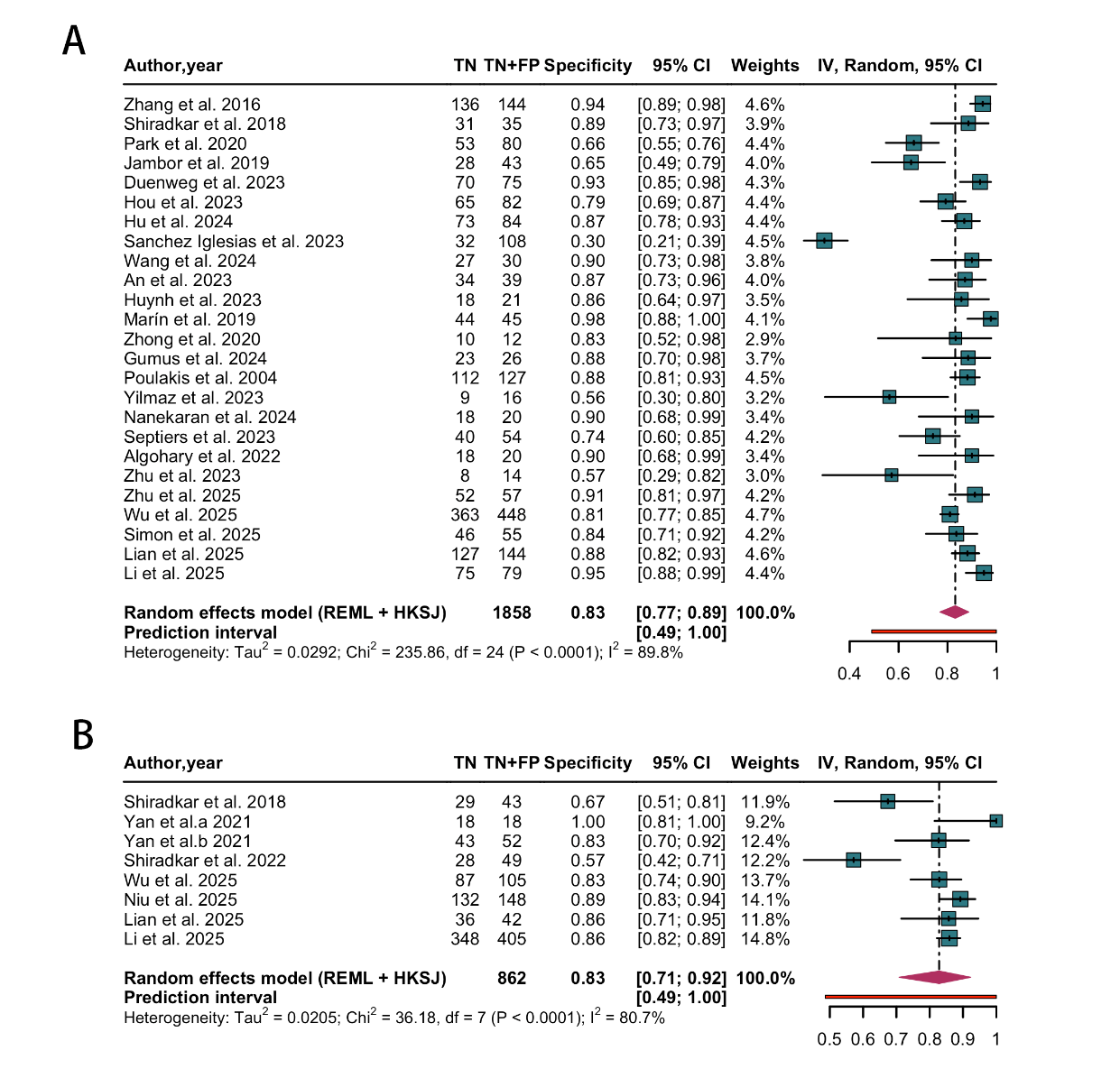


### Figure S3. Forest Plots of Diagnostic Odds Ratio (DOR): (A) internal validation sets and (B) external validation sets.


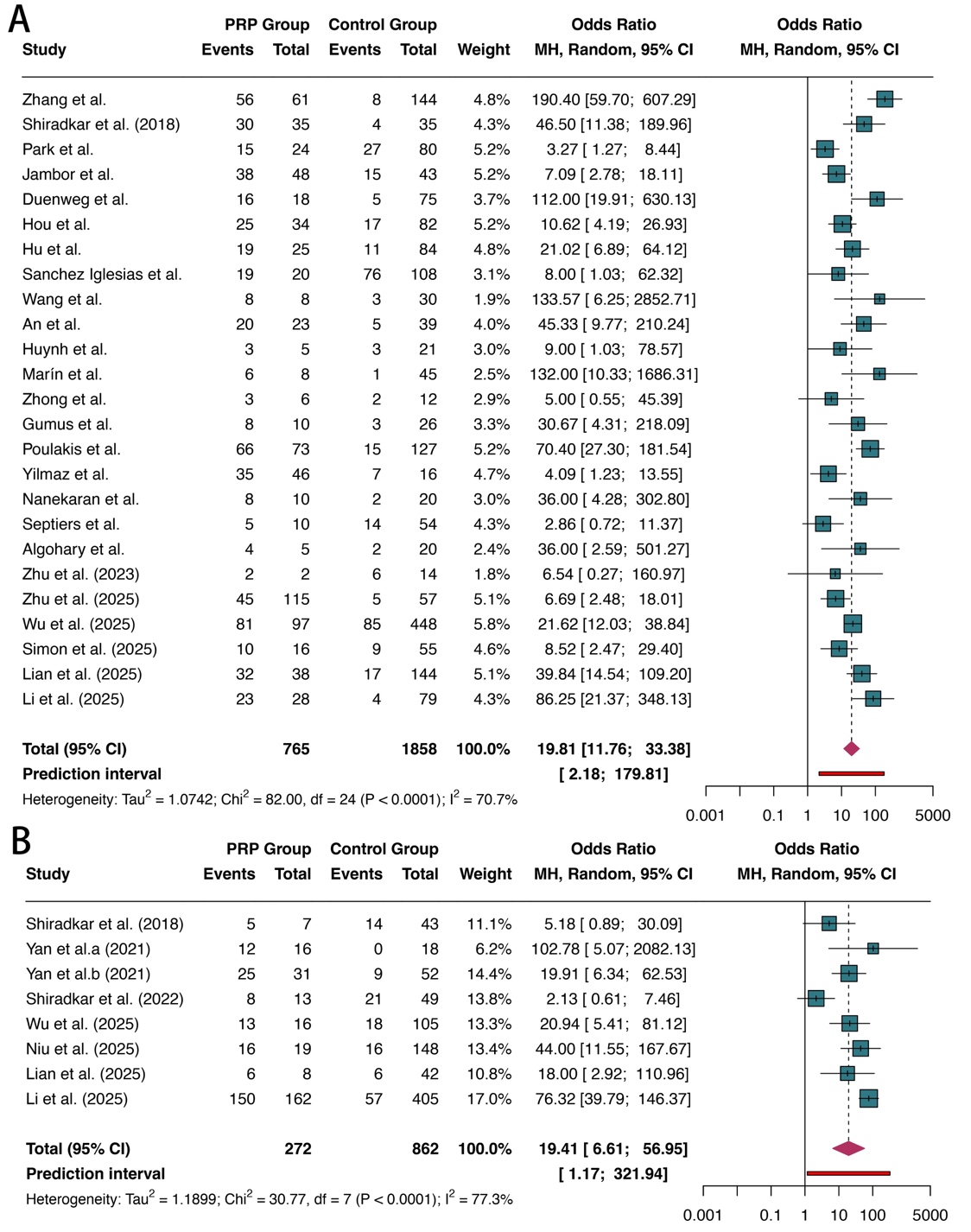


### Figure S4. Fagan plots for assessing clinical applicability: (A) internal validation sets and (B) external validation sets.


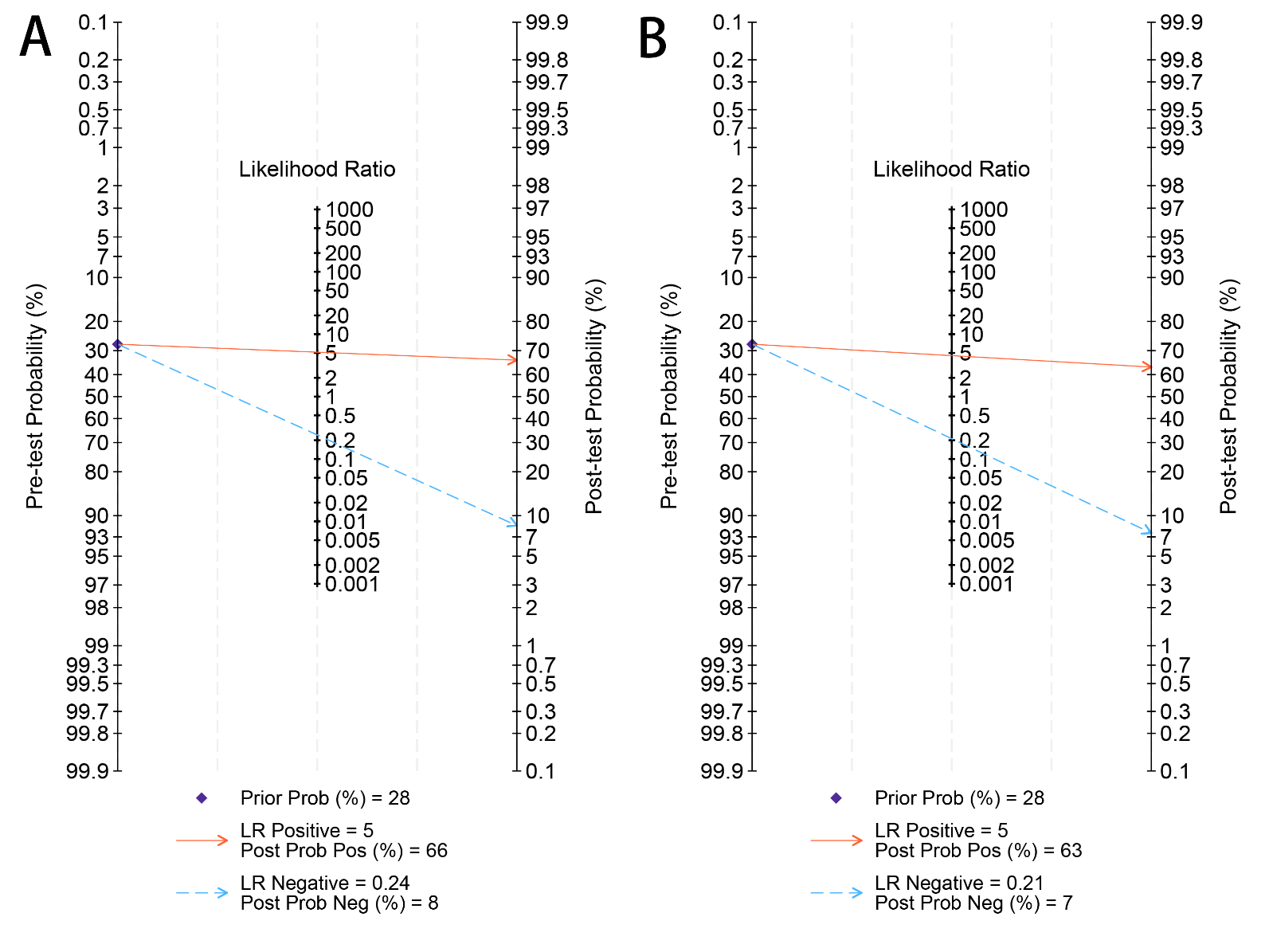


### Figure S5. Deeks’ Funnel plot asymmetry test for evaluating publication bias: (A) internal validation sets and (B) external validation sets.


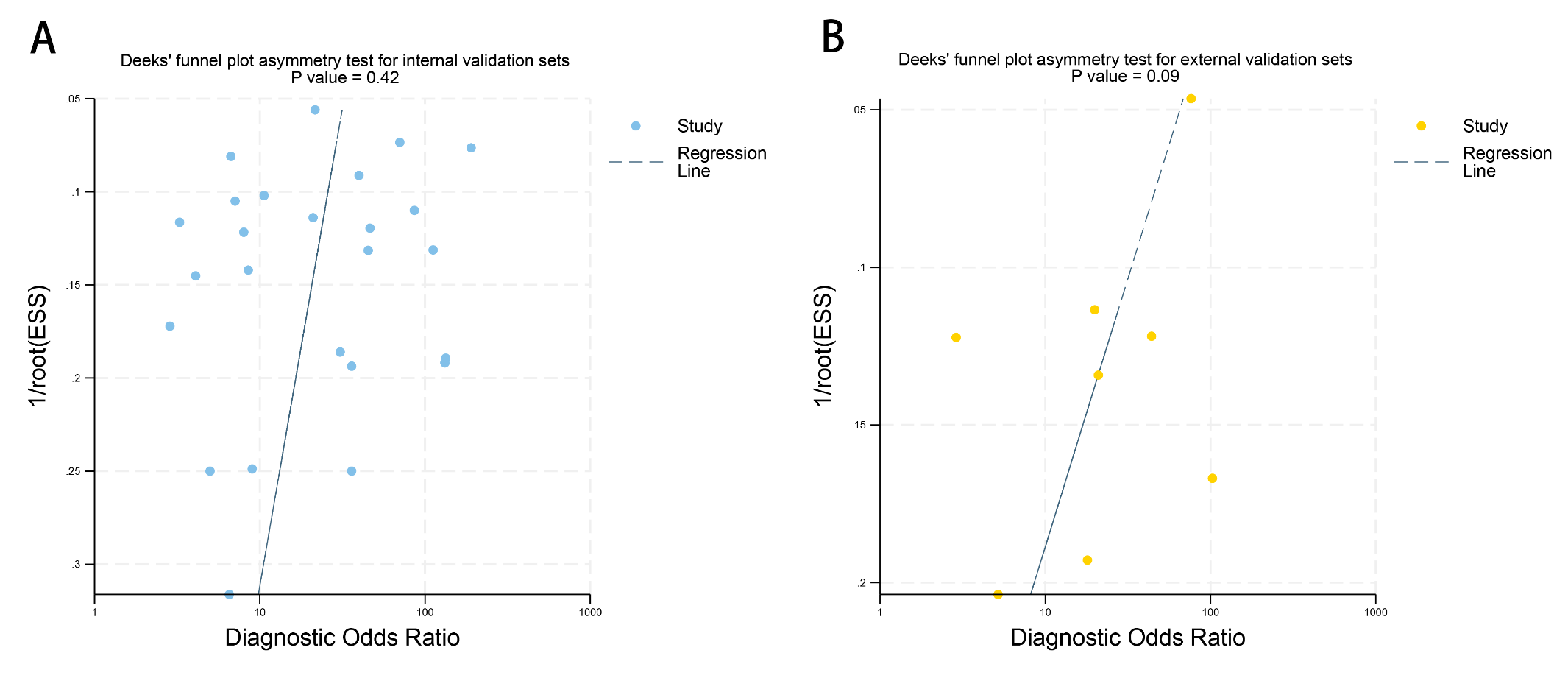

Supplement: Multimedia Appendix 1 [file jmir-v28-e85360-s001.docx]
