# Supplementary material for: Magnetic Resonance Imaging–Based Artificial Intelligence in Predicting Prostate Cancer Biochemical Recurrence: Systematic Review and Meta-Analysis
Source: J Med Internet Res. 2026 Jul 7;28:e85360. doi: 10.2196/85360 (PMC13340575; doi:10.2196/85360)
Supplement: Checklist 4 [file jmir-v28-e85360-s005.docx]

### Supplementary Table 4. Compliance with the CHARMS (CHecklist for critical Appraisal and data extraction for systematic Reviews of prediction Modelling Studies) framework. This table maps each CHARMS domain to the corresponding content and location in the manuscript, describing how data extraction and critical appraisal were conducted for all 28 included studies of MRI-based AI models predicting biochemical recurrence in prostate cancer.

| **CHARMS Domain** | **Key Items to Extract (per CHARMS)** | **How This Review Addresses the Domain** | **Location in Manuscript** |
| --- | --- | --- | --- |
| **1. Source of Data** | Design (e.g., cohort, case-control, RCT); data source (e.g., registry, hospital); setting (single vs. multi-center) | 26/28 studies were retrospective; 2 prospective. Data were sourced from institutional hospital databases (25 studies) and public datasets (3 studies). 13 studies originated from China, with remaining studies from USA, Europe, Korea, Canada, and Iran. Single-center designs predominated; 7 studies included multi-center external validation. | Methods: Inclusion and Exclusion Criteria; Results: Study Description and Quality Assessment; Table 1 |
| **2. Participants** | Participant recruitment method; inclusion/exclusion criteria; treatment received; representative of target population | Participants were adults with pathologically confirmed prostate cancer (PCa) who underwent radical prostatectomy (RP, 17 studies), radiation therapy (RT) with or without hormone therapy (HT, 11 studies), including EBRT alone (3), EBRT+HT (3), and brachytherapy (1). Total 2,623 patients in internal validation cohorts and 1,134 patients in external validation cohorts. Patient-based analysis in 26/28 studies; lesion-based in 2. | Methods: Inclusion and Exclusion Criteria (PITROS framework); Results: Study Description; Table 1 |
| **3. Outcome(s) to be Predicted** | Definition and method of measurement of outcome; whether the outcome was assessed blinded; time of outcome measurement | Outcome: biochemical recurrence (BCR), dichotomized as BCR-positive vs. BCR-negative. Definitions included AUA (16 studies), Phoenix (9 studies), combined AUA+Phoenix (1), Phoenix+CRPC (1), and study-specific PSA ≥0.1 ng/ml (1). BCR assessment was based on serial post-treatment PSA measurements per each definition. Heterogeneity in BCR definitions was noted and addressed via treatment-stratified subgroup analyses. | Methods: Inclusion Criteria; Results: Study Description; Discussion (limitation 3) |
| **4. Candidate Predictors (Index Tests)** | Type and number of predictors; method and timing of predictor measurement; blinding of predictor assessment to outcome | Predictors: MRI-based radiomic features extracted from T2WI, DWI, ADC, and/or DCE sequences. 10 studies used radiomics-only models; 18 studies combined radiomic features with clinical variables (e.g., PSA, Gleason score, T-stage). MRI acquisition was pre-treatment in 24 studies, post-treatment in 3, and both in 1. Blinding of predictor assessment to outcome was assessed via PROBAST+AI (predictor domain). | Methods: Data Extraction; Results: Study Description; Table 2; Suppl Tables 5–6 (PROBAST+AI predictor domain) |
| **5. Sample Size** | Number of participants and events; events per variable (EPV); power or sample size justification | Sample sizes ranged from n=10 (Zhong et al. 2020, internal validation) to n=811 (Wu et al. 2025, training set). Internal validation cohorts: median sizes reported in Table 1. External validation cohorts: 7 studies with 8 datasets, ranging from n=34 to n=753. Several studies had small sample sizes (e.g., Gumus n=36, Zhong n=18), noted as a limitation increasing overfitting risk. No included study explicitly reported EPV or sample size justification; this was captured in the PROBAST+AI analysis domain. | Results: Study Description; Table 1; Discussion: Limitations (para 2); Suppl Tables 5–6 |
| **6. Missing Data** | Handling of missing data (e.g., complete case, imputation); number and percentage of missing data for predictors and outcome | The majority of included studies did not report details on missing data handling. This deficiency was captured in the PROBAST+AI quality assessment: the analysis domain (signaling question 4.3: "Were participants with missing or censored data handled appropriately in the analysis?") rated several studies as unclear (U) or high (H) risk. This is acknowledged as a limitation of the primary evidence base. | Results: Quality Assessment; Suppl Tables 5–6 (domain d, item 4.3); Discussion: Limitations |
| **7. Model Development** | Modeling method; predictor selection method; any internal validation used during development (e.g., cross-validation, bootstrap); handling of model complexity | 19 studies employed machine learning (logistic regression in 5/21 [24%], SVM, XGBoost, random forest, etc.); 9 studies used deep learning (SNN, CNN, Transformer). Feature selection methods varied. Internal validation strategies included data splitting (train/test), k-fold cross-validation, and leave-one-out. Overfitting handling assessed via PROBAST+AI analysis domain (item 4.5). Radar charts illustrate algorithm distribution (Figure 3). | Methods: Data Extraction; Results: Study Description, Diagnostic performance of different AI algorithms; Table 2; Figure 3; Suppl Tables 5–6 |
| **8. Model Performance** | Discrimination (e.g., c-statistic/AUC); calibration; classification measures (sensitivity, specificity); overall diagnostic performance | Discrimination: pooled AUC 0.86 (95% CI: 0.83–0.89) internal, 0.84 (0.79–0.90) external. Sensitivity: 0.80 (0.73–0.86) internal, 0.82 (0.72–0.91) external. Specificity: 0.83 (0.77–0.89) internal, 0.83 (0.71–0.92) external. DOR: 19.81 (11.76–33.38) internal, 19.41 (6.61–56.95) external. Prediction intervals and Tau² reported for all pooled estimates. Calibration data were not systematically reported in primary studies; noted as a limitation. | Results: Internal and external validation sets; Suppl Figures 1–3; Figure 6; Table 3 |
| **9. Model Evaluation** | Method of evaluation (internal vs. external validation); comparison across datasets; evaluation of heterogeneity and clinical utility | Internal validation: 25 studies (25 datasets). External validation: 7 studies (8 datasets). Z-tests compared performance between internal and external validation (no significant differences: P=0.73, 1.00, 0.53, 0.98 for sensitivity, specificity, AUC, DOR). Risk of bias assessed using PROBAST+AI for both model development and evaluation phases. Clinical utility evaluated via Fagan nomogram. Small-study effects assessed via Deeks’ funnel plot. | Methods: Quality Assessment, Statistical Analysis; Results: all subsections; Suppl Figures 4–5; Suppl Tables 5–7 |
| **10. Results** | Final prediction model presentation; subgroup analyses; heterogeneity exploration; sensitivity analyses | Subgroup analyses by AI algorithm type, treatment modality (RP, EBRT±HT, BT), MRI timing (pre/post-treatment), and AI model type (radiomics-only vs. radiomics+clinical). Meta-regression identified AI method, model type, MRI timing, and treatment as potential sources of heterogeneity. Bivariate box plots identified outlier studies. Bubble plots evaluated temporal trends in DOR. All treatment-stratified results described as hypothesis-generating. | Results: Subgroup analysis, Bivariate box plots and meta-regression, Clinical utility; Figures 4–8; Table 3 |
| **11. Interpretation and Discussion** | Interpretation of results (confirmatory vs. exploratory); comparison with existing models/reviews; clinical implications; limitations; recommendations for future research | Results interpreted as exploratory/hypothesis-generating. AI models described as "promising investigational tools" not yet ready for routine clinical use. Compared with Liu et al. (2024) and Salimi et al. (2025). Sensitivity-specificity trade-off discussed for radiomics+clinical models. Pre-treatment MRI identified as superior for specificity. Limitations: retrospective designs, small samples, heterogeneous BCR definitions, optimistic bias from best-model selection, limited interpretability, and lack of comparison with radiologists. Future directions: prospective multicenter studies, standardized MRI protocols, federated learning. | Discussion (all paragraphs); Conclusion |
